# Competition among networks highlights the power of the weak

**DOI:** 10.1038/ncomms13273

**Published:** 2016-11-14

**Authors:** Jaime Iranzo, Javier M. Buldú, Jacobo Aguirre

**Affiliations:** 1 National Center for Biotechnology Information, National Library of Medicine, National Institutes of Health, Bethesda, Maryland 20894, USA; 2 Laboratory of Biological Networks, Center for Biomedical Technology (UPM), Pozuelo de Alarcón, 28223 Madrid, Spain; 3 Complex Systems Group, Universidad Rey Juan Carlos, Móstoles, 28933 Madrid, Spain; 4 Grupo Interdisciplinar de Sistemas Complejos (GISC), Madrid, Spain; 5 Centro Nacional de Biotecnología, CSIC. c/Darwin 3, 28049 Madrid, Spain

## Abstract

The unpreventable connections between real networked systems have recently called for an examination of percolation, diffusion or synchronization phenomena in multilayer networks. Here we use network science and game theory to explore interactions in networks-of-networks and model these as a game for gaining importance. We propose a viewpoint where networks choose the connection strategies, in contrast with classical approaches where nodes are the active players. Specifically, we investigate how creating paths between networks leads to different Nash equilibria that determine their structural and dynamical properties. In a wide variety of cases, selecting adequate connections leads to a cooperative solution that allows weak networks to overcome the strongest opponent. Counterintuitively, each weak network can induce a global transition to such cooperative configuration regardless of the actions of the strongest network. This power of the weak reveals a critical dominance of the underdogs in the fate of networks-of-networks.

## Abstract (web summary)

Network science and game theory have been traditionally combined to analyse interactions between nodes of a network. Here, the authors model competition for importance among networks themselves, and reveal dominance of the underdogs in the fate of networks-of-networks.

Social, biological, physical and technological systems are composed of a diversity of interacting agents, leading network science, a statistical physics understanding of graph theory, to be a genuine tool for investigating their structure and dynamics^1^,^2^,^3^. Within the framework of social networks^4^, the topology of the interactions between individuals has been demonstrated to be crucial in, for example, the vanishing of the critical threshold in epidemics^5^,^6^ or the efficient and fast propagation of innovation^7^. In a similar manner, the topology of a network itself can be influenced by the dynamical processes occurring in it, giving rise to adaptive mechanisms that rule the evolution of the structure of social networks^8^.

The emergence of cooperation, defection or altruism can be investigated by linking game theory to network science^9^,^10^,^11^,^12^. In this way, the intrinsic heterogeneity of social networks, the majority of them showing power-law distributions in the number of connections^1^, has been related in many cases to the emergence of cooperation, contrary to what is observed in homogeneous populations^13^. Furthermore, highly connected individuals have also been shown to be more prone to collaborate than scarcely connected ones^14^. Under this framework, the understanding of evolutionary games largely benefited from the methodological tools of network science^11^. Although attention was initially focused on the interplay between nodes' strategies and the structure of the underlying (single) network^15^, more recently, coevolutionary rules have also been related to the emergence of interdependency^16^ and multilayer structures^17^. However, what if we are concerned about the interests of a network as a whole instead of its nodes? Does it make sense to consider networks competing or collaborating with other networks? The fruitful recent literature about networks-of-networks, or in a more general context about multilayer networks, makes these two questions timely and extremely relevant^18^,^19^. A diversity of dynamical processes such as percolation^20^, diffusion^21^ or synchronization^22^ have been recently reinterpreted by assuming that real networks unavoidably interact with other networks, a contact that may be beneficial or detrimental to each of the networks belonging to the ensemble^23^.

Here we investigate how *m*>2 networks compete or cooperate to achieve a relative increase of importance measured as eigenvector centrality, which maximizes their outcome in a variety of dynamical processes. In our competition, networks can vary the way they interact with other networks, evolving in time until they reach a stable situation where all networks refuse to modify their strategy, because any change would lead to a worse result. Importantly, an a priori optimal connection strategy for a given network may not be reachable due to the actions of the competitor networks, which turns the analysis of the final outcome of the networks into a study of Nash equilibria^24^ in a network-of-networks. With this objective in mind, we define a methodology to analyse the competition among networks of any size or topology, demonstrating that several Nash equilibria can coexist, with some of them benefiting the strongest networks and others benefiting the weaker ones.

In particular, we report the existence of a wide regime of the system parameters in which every weak network can induce the rest to cooperate, to escape from a detrimental Nash equilibrium, taking over the final situation of the whole network-of-networks. Paradoxically, the strong network cannot reverse this phenomenon. This counter-intuitive asymmetry that promotes the cooperation among weak networks is independent of the network structure or the competition rules and it could be applicable to an extensive number of real systems.

## Results

### Defining the rules of the competition

**Figure 1:**
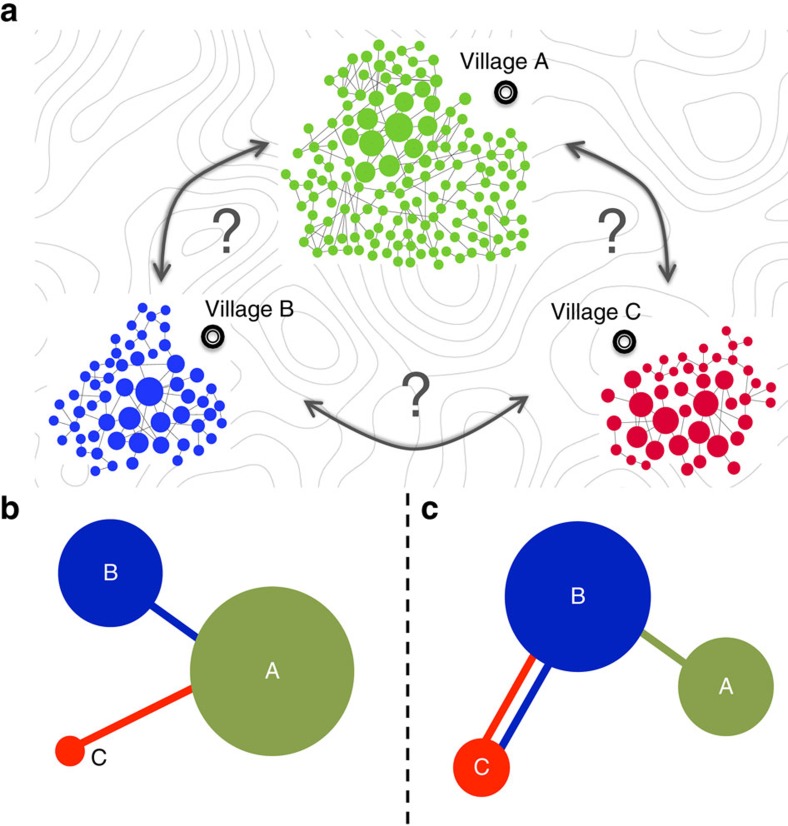
Competition for centrality among loan networks. (**a**) Loan networks of three independent Indian villages, from refs 25, 26. Villages A (green), B (blue) and C (red) are named according to the largest eigenvalue of their loan networks, such that *λ*_A_>*λ*_B_>*λ*_C_ (see Supplementary Note 1 for details about the construction of the networks). Creating connections between villages would lead to a network-of-networks T, whose centrality is distributed among the villages. In **b**,**c**, we show the centrality retained at each village (network) depending on different connection strategies. The radius of each circle is proportional to the centrality accumulated by each network. Networks' strengths are *λ*_A_=4.27, *λ*_B_=4.05 and *λ*_C_=3.38. When one connection between villages is allowed (*l*=1), two Nash Equilibria coexist: in **b**, networks B and C connect to A (*C*_A_=0.55, *C*_B_=0.35 and *C*_C_=0.10), but their best strategy is shown in **c**, that is, to create links between them, forcing network A to join them (*C*_A_=0.32, *C*_B_=0.49 and *C*_C_=0.19). In summary, the final result of the contest is largely dependent on the reached solution.

As a general rule, we consider that nodes belonging to a network will accept a common strategy, which can be justified in terms of a common benefit or the existence of an imposition within a hierarchical organization (see Supplementary Note 1 for details). The following example, based on real networks, illustrates how different strategies can enhance the outcome of a network. The interaction between members of rural communities in southern India was recently investigated through a series of surveys in the framework of a microfinance programme^25^,^26^. From those data sets (available at the online version of ref. 25), we constructed the loan networks inside three of those villages (see Fig. 1a), creating a link between individuals *i* and *j* if they were willing to lend to or borrow from each other a certain amount of money. Local loan networks constructed as explained above provide much information about the financial resilience of a region. If the local authorities of one village promoted the connections to other regions—for example, through the funding of social events—their loan network would be enhanced and the village would become more prepared to face unexpected natural or financial risks (see refs 27, 28, 29, 30, 31 and Supplementary Note 1). However, what village is the best to connect to if more than one option exists? In addition, more importantly, which village would benefit the most from the creation of new financial channels among them?

To address these questions in a general framework we consider *m* networks of *N*_*i*_ nodes respectively, where *i*=A, B, C,... The adjacency matrices **G**_*i*_ associated to each network *i* contain the full information about the connections between their nodes (that is, the specific topology of the networks). The largest eigenvalue *λ*_*i*_ of **G**_*i*_ is an indicator of the network strength, as explained in ref. 32; thus, we can order them such that *λ*_A_>*λ*_B_≥ ... ≥*λ*_*m*_. We make use of the eigenvector centrality to determine the importance acquired by each node, which is directly obtained from the eigenvector **u**_1_ associated with the largest eigenvalue *λ*_1_ of the adjacency matrix (see ref. 3, Methods and Supplementary Note 2).

In our game, every competitor (that is, network) makes use of up to *l* undirected links to connect with any of the other networks. The connector nodes are those with the largest centrality (see Methods). The refusal to connect is an accepted strategy. Therefore, there are 
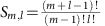
 strategies per competitor and (*S*_*m*,*l*_)^*m*^ possible combinations of actions. The target of each competitor is to maximize its own eigenvector centrality, calculated as the total importance (or centrality) *C*_*i*_ accumulated by all its nodes


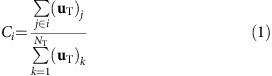


where *j* are the nodes that belong to network *i* and **u**_T_ is the eigenvector associated with the largest eigenvalue *λ*_T_ of the adjacency matrix of the network-of-networks T, containing all nodes *N*_T_=∑_*i*_ *N*_*i*_. It is noteworthy that we face a zero-sum game (∑_*i*_ *C*_*i*_=1) and the connected system T consists of *m* networks interconnected through a maximum of *m* × *l* connector links. Furthermore, such connector links will influence *C*_*i*_ of every network, but not their strength *λ*_*i*_, which is measured when network *i* is isolated from the rest and is independent of the contest.

As we assume that networks are able to modify their connector links with the aim of acquiring the highest possible centrality *C*_*i*_ inside the network-of-networks T, the final configuration of the system is given by a Nash equilibrium. A Nash equilibrium is the solution of a non-cooperative game involving two or more players, in which each competitor is assumed to know the equilibrium strategies of the other players and no competitor has anything to gain by changing only their own strategy^24^,^33^. From this general perspective, regardless of the particular rules of the contest, the competition process finishes when a Nash equilibrium is reached, being *C*_*i*_ the final payoff of each competitor network.

Figure 1b,c show the competition for centrality in our ‘tale of three villages'. We compute the largest eigenvalue associated with the loan networks within each village and obtain the ranking in strength: *λ*_A_>*λ*_B_>*λ*_C_. The terminology used to indicate how a network (that is, a village) *i*_1_ decides to connect to a network *i*_2_ is the following: *i*_1_(0) stands for network *i*_1_ refusing to connect, *i*_1_→*i*_2_ stands for *i*_1_ connecting to network *i*_2_ and *i*_1_↔*i*_2_ stands for *i*_1_ connecting to network *i*_2_ and *i*_2_ connecting to network *i*_1_. The arrow → indicates which network decides to create the connector link, but all links are undirected (that is, bidirectional).

Allowing villages to connect through one link (*l*=1), we obtain two possible Nash equilibria. In one of those, weak villages establish connections with the strong village, {A(0), B→A, C→A}, which plainly benefits the latter (Fig. 1b). Nevertheless, the alternative equilibrium {A→B, B↔C} allows weak villages to overcome their stronger competitor by connecting with each other. In this scenario, the strong network must connect to B to retain part of the centrality of the whole system (Fig. 1c).

Importantly, the selection of adequate connection strategies goes beyond the competition for centrality. In professional networks, for example, the growth of knowledge of an individual can be modelled to be proportional to the knowledge of his/her acquaintances^34^, which leads to a final distribution of knowledge that is given by the first eigenvector **u**_T_ of the adjacency matrix. Translated to a case where independent groups of professionals or researchers can create connections among them, this would indicate that the strategy showed in Fig. 1c would enhance not only the importance of a cluster of professionals, but the relative amount of knowledge acquired by the weakest group compared with the strongest one (see Supplementary Note 1 for several real-world examples dealing with social, technological and biological networks). Furthermore, a wide variety of systems are described by weighted adjacency matrices—transition matrices that include the specificities of the underlying dynamical process—whose vector **u**_T_ is related to the equilibrium state of the system^32^. Replication-mutation evolutionary dynamics^35^, diffusion processes^21^ or disease spreading^36^ are only a few examples where the methodology presented here can be applied with no loss of generality (see Methods).

### Competition and cooperation to overcome the strongest

**Figure 2:**
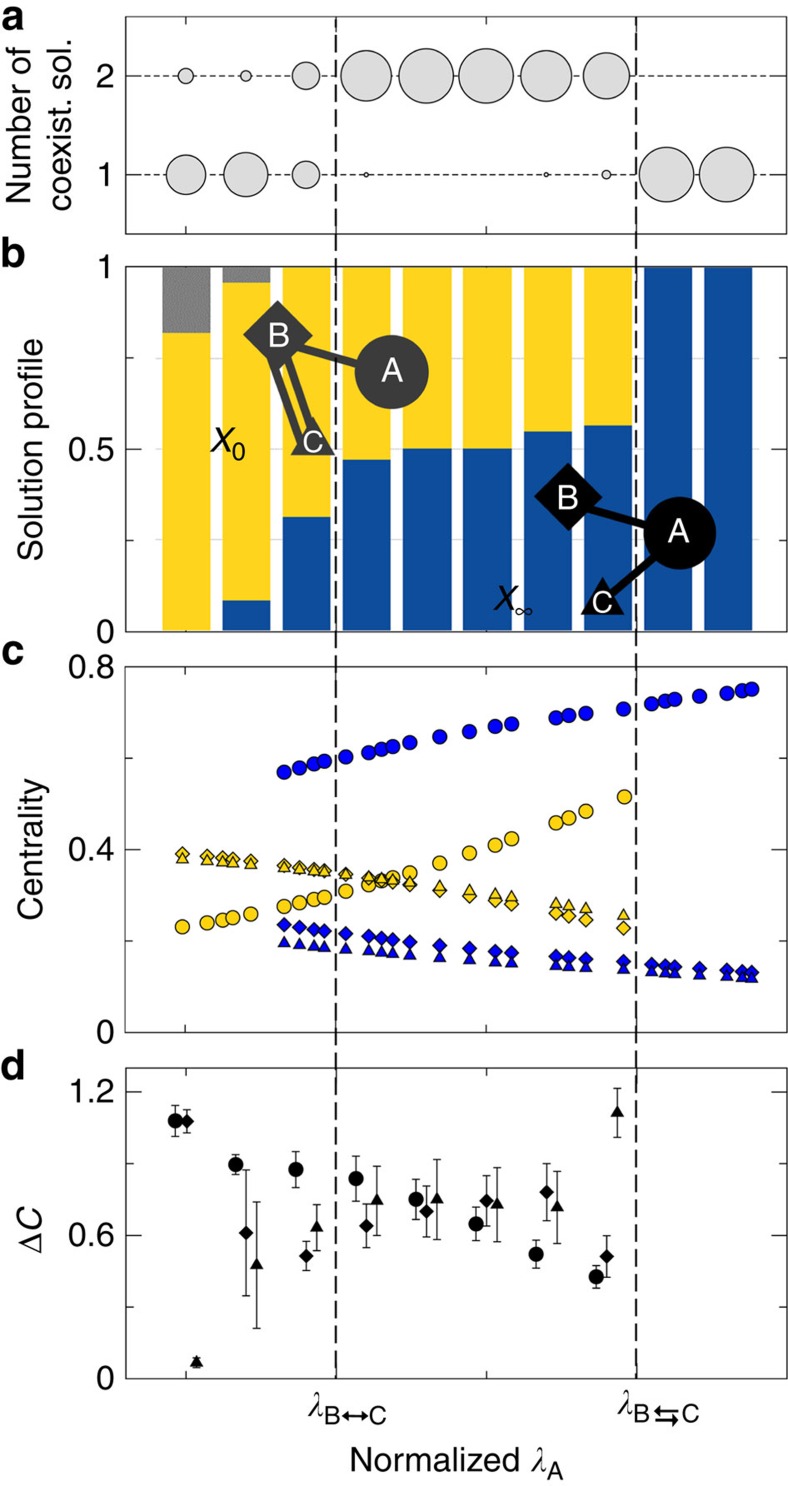
Competition for centrality among 3 networks. Each competitor makes use of as much as *l*=1 link to connect with the rest of the networks. We modify the size and/or mean degree of network A (that is, the strongest competitor) in order to increase its strength from *λ*_A_=*λ*_B_ to *λ*_A_≫*λ*_B↔C_, where B↔C is the network resulting from connecting B and C through a double link. The *x* axis has been rescaled to allow for comparisons among different realizations. For each choice of B and C, the system is solved for 20 series of A and results are an average of more than 500 sets of A, B and C. (**a**) Number of coexisting Nash equilibria per realization. The radius of each circle is proportional to the fraction of realizations (no cases with more than two coexisting solutions were found). (**b**) Relative occurrence of different configurations in the set of solutions, averaged over all realizations: (i) Equilibrium *X*_0_={A→B, B↔C} (yellow), (ii) equilibrium *X*_∞_={A(0), B→A, C→A} (blue) and (iii) other equilibria (grey). In some exceptional realizations, A→B is substituted by A→C in *X*_0_. (**c**) Centrality of networks A (circle), B (diamond) and C (triangle) for a particular choice of B and C (*λ*_B_=5.25 and *λ*_C_=5.2). The results are shown for solutions *X*_0_ and *X*_∞_ (colour code as in **b**). (**d**) Relative centrality variability Δ*C* among different Nash equilibria. Data points (error bars) correspond to averages (s.d.) over all realizations whose *λ*_A_ lies in the corresponding X-axis interval. Network symbols as in **c**.

Figure 2 shows a complete numerical description of the competition among *m*=3 generic scale-free networks A, B and C of largest eigenvalues *λ*_A_>*λ*_B_>*λ*_C_. For the sake of clarity, only one connector link is allowed per network (*l*=1) and, therefore, each competitor has *m* different connection strategies (that is, connecting to any of the other *m*−1 networks or refusing to connect). In the case of three networks, 27 combinations of strategies are possible for each realization—choice of networks—among which only those that verify the conditions to be Nash equilibria are taken as solutions to the contest. Figure 1b,c show that more than one final solution can coexist, which poses two relevant questions: (i) is the coexistence of solutions a general phenomenon? And if this is the case, (ii) does the final outcome of each player vary substantially depending on the solution reached?

Figure 2a,b address question (i) and show the solution profiles as the strength of network A (that is, *λ*_A_) is increased. Figure 2a shows a scenario that we found to be general: the coexistence of Nash equilibria, among which two of them, called *X*_0_ and *X*_∞_, are especially relevant (see Fig. 2b):


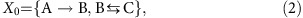



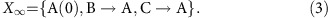


On the other hand, Fig. 2c shows the centrality for the two existing Nash equilibria as the strength of A is increased for a particular choice of B and C. For a wide range of values of *λ*_A_, the centralities attained by each player strongly depend on the specific solution of the contest, which answers question (ii) and highlights the importance of choosing an adequate connection strategy.

To check the relevance of this result, in Fig. 2d we show the relative centrality variability (Δ*C*), a measure of how much a player outcome improves by getting to its optimal solution:


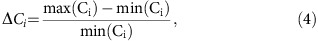


where the maxima and minima are computed among all coexisting Nash equilibria. The Δ*C*_*i*_ of each player *i* takes values ranging from zero (if all solutions lead to the same centrality) to infinity (if the worst-case solution leads to zero centrality for that player). All three networks show values of the order of Δ*C*∼0.8, which means that the final centrality of each competing network can vary up to almost twofold depending on the solution reached.

In view of all, we can identify different competition regimes depending on the relative strength between the strong network A and the rest of competitors. For very large strengths of A, that is, when *λ*_A_>*λ*_B↔C_, the only existing Nash equilibrium corresponds to *X*_∞_: small networks avoid mutual cooperation and tend to connect to the largest one, which dominates the contest. A critical transition occurs at *λ*_A_=*λ*_B↔C_ below which *X*_∞_ and *X*_0_ coexist in a bistable manner. Interestingly, within this region, cooperation between the weak networks always leads to their best outcome. Finally, a smoother transition occurs around *λ*_A_∼*λ*_B↔C_, being B↔C the network resulting from connecting B and C through a single link: when the strength of A approaches that of B, mutual cooperation between B and C becomes dominant and equilibrium *X*_∞_ is no longer possible. At the same time, other Nash equilibria may appear (grey region of Fig. 2b). See Supplementary Notes 2 and 3 for a full analytical treatment of this phenomenon.

### Weak networks can induce migration between equilibria

**Figure 3:**
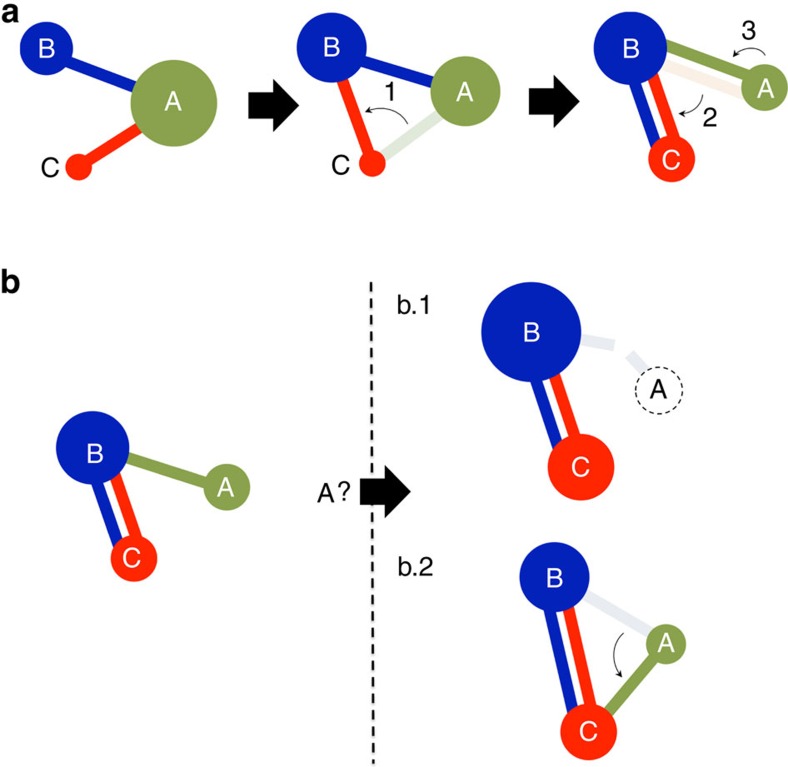
Migration between Nash equilibria. In this example, networks are generated with the Barabási–Albert model (*λ*_A_=4.07, *λ*_B_=3.95 and *λ*_C_=3.63, yielding *λ*_A_<*λ*_B↔C_=4.21). To follow how the connection strategies influence the distribution of centrality, the radius of each circle is proportional to the centrality accumulated by each network. Equilibrium *X*_∞_ leads to *C*_A_=0.65, *C*_B_=0.21 and *C*_C_=0.14, while *X*_0_ leads to *C*_A_=0.28, *C*_B_=0.45 and *C*_C_=0.27. (**a**) The weakest network C provokes the migration from *X*_∞_ to *X*_0_, to drastically improve its centrality (the same reasoning could be applied to B). Step 1: network C disconnects from the strong network A and connects to the weak network B. Step 2: A does not change its connections because any variation would be detrimental, but B improves its centrality detaching from A and connecting to C. Step 3: A becomes isolated and *C*_A_=0 (because *λ*_A_<*λ*_B↔C_). It is obliged to connect to B and the system reaches the Nash equilibrium *X*_0_. (**b**) The strong network A cannot provoke the migration from equilibrium *X*_0_ to *X*_∞_ and it is obliged to stay in a disadvantageous final state. If either A refuses to connect to any weak network (Step b.1) or connects to C instead (Step b.2), B and C would lose centrality if they broke their mutual connection and consequently refuse to change their connections. A becomes obliged to connect again to B returning to strategy *X*_0_.

As explained, in a Nash equilibrium every competitor that may change strategy would decrease its centrality. However, as there are coexisting Nash equilibria, it may be worth assuming a temporal loss of centrality if the final situation leads to an improvement in the outcome. In this way, we study the potential migration between equilibria *X*_0_ and *X*_∞_ in the regime in which they coexist (*λ*_A_<*λ*_B↔C_). In Fig. 3a we show that the migration from *X*_∞_ to *X*_0_ can be provoked by the weakest network C individually, whereas Fig. 3b shows that the strong network cannot trigger the system to migrate from *X*_0_ to *X*_∞_. As a consequence, when multiple networks compete for centrality, a weak network by itself might escape from a detrimental equilibrium and push the whole system towards a much more beneficial one with the cost of a one-step transient during which its centrality is decreased. On the contrary, this migration-strategy is not accessible to the strong network, resulting in a natural asymmetry in the context of networks-of-networks that benefits the final outcome of weak networks and provides them with a flexibility not permitted to the strongest competitors.

### Overall consequences on the network-of-networks

 Extensive numerical work yields that the strength *λ*_T_ of the network-of-networks T following the equilibrium *X*_0_ is always larger than that of the solution *X*_∞_ (that is, *λ*_T_(*X*_0_)>*λ*_T_(*X*_∞_), see Supplementary Note 4). Importantly, an increase of *λ*_T_ is related to enhanced growth at the equilibrium for a wide range of dynamical processes^35^, a reduction of the critical coupling strength for the emergence of synchronization (as ∼1/*λ*_T_)^37^ or the emergence of a giant component in percolation phenomena^38^. Therefore, the natural tendency towards cooperation among weak networks presented in this work also improves efficiency and growth of the whole system. Going back to the three villages example, the analysis of the Nash equilibria reveals that the *X*_0_ equilibrium (Fig. 1c) leads to a higher global *λ*_T_ than *X*_∞_ (Fig. 1b; that is, *λ*_T_(*X*_0_)=4.78>*λ*_T_(*X*_∞_)=4.57, for *l*=1). At the end, this is good news for all villages, as a higher *λ*_T_ enhances the strength of the whole ensemble^39^. It is noteworthy that these results can be used not only for description but, more importantly, for prescription of how networks can maximize their outcome when interacting with other networks and how the appearance of new interactions among isolated networks influences the structural and dynamical properties of real systems.

Finally, the migration between equilibria described above could have a suggestive counterpart in a wide variety of situations where a relationship based on subjugation to a powerful leader naturally migrated towards a new and more productive model based on cooperation (see refs 40, 41 and Supplementary Note 4 for an illustrative historical example).

## Discussion

In summary, we propose combining network science and game theory to analyse the election of interconnecting strategies in a zero-sum game where players are not single agents but networks. The creation of paths between interacting networks leads to different Nash equilibria, some of them benefiting the strong competitor and some of them reinforcing the underdogs. Counterintuitively, we showed that transitions between coexisting Nash equilibria are restricted to the weakest competitors, which in practice rule the contest, whereas the strongest network is unable to change the *status quo* in its own interest.

Importantly, most assumptions of our model can be modified to describe more realistic scenarios without causing qualitative changes (see Supplementary Note 5 for details). When each player is permitted to connect to the rest of networks through more than one link (that is, *l*>1), the number of strategy combinations grows as *l*^*m*(*m*−1)^ for fixed *m*. However, the number of coexisting solutions and the phenomenology observed are totally equivalent to those obtained for *l*=1. The same applies for random (Erdös–Rényi) network topologies, networks of any size and networks with different abilities—that is, when certain networks can connect through a larger number of connector links (or even more weighted links) than the other competitors.

Furthermore, extending the analysis to mixed Nash equilibria, where any non-integer distribution of the *l* connector links is allowed, does not alter the results and provides a probabilistic nature to the game that broadens its applicability. When more than 3 networks are considered in the contest, new kinds of Nash equilibria arise, but again, we observe the existence of wide regions of the parameter space where weak networks rule the whole network-of-networks. In addition, different definitions of the centrality-based payoffs have been analysed—such as betweenness or closeness centrality—and only those closely related to the eigenvector centrality lead to a rich phenomenology in the number of Nash equilibria and the effect of such equilibria on the payoffs of the competitors.

Finally, in some social and economical networks the connection strategies may be influenced by the individual motivations of the connector nodes, resulting in a potential conflict with the collective interests. As a first step in understanding such complex scenarios, we introduced a payoff following ref. 42, which includes both individual and collective contributions (see Supplementary Note 6): we conclude that cooperation between the weak networks and their control of the game is a frequent outcome that can appear at relatively small (or even zero) levels of collective incentive, although the quantitative details significantly depend on the topology of the networks.

The robustness of the phenomenology presented here, added to its potential applicability to real cases, makes this ‘power of the weak' a valuable fact to consider in future modelling of technological, biological or sociological processes on networks.

## Methods

### Measuring the importance of nodes and networks

We use the eigenvector centrality *x*_*k*_ for quantifying the importance of a node *k* in a network, which can be obtained as an iterative process that sums the centralities of all neighbours of *k*:


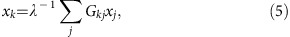


where *λ* is a constant, *x*_*k*_ is the eigenvector centrality of node *k* and *G*_*kj*_ are the components of the adjacency matrix, which could be both binary or weighted^43^. In matrix notation equation (5) reads *λ***x**=**Gx** so that **x** can be expressed as a linear combination of the eigenvectors **u**_*k*_ of the adjacency matrix **G**, being *λ*_*k*_ the set of the corresponding eigenvalues. Equation (5) can be regarded as an iterative process that begins at *t*=0 with a set of initial conditions **x**_0_. Regardless of the values of **x**_0_, the value of **x**(*t*) at *t*→∞ will be proportional to the eigenvector **u**_1_ associated with the largest eigenvalue *λ*_1_. Therefore, the eigenvector centrality is directly obtained from the eigenvector **u**_1_ of the adjacency matrix **G**, which also holds for weighted adjacency matrices. As explained in the main text, the centrality accumulated by each network is obtained as the fraction of centrality accumulated by their nodes. Finally, we use *λ*_1_ as the measure of the network strength, as it is related to a series of dynamical properties of networks and, in turn, it increases with the number of nodes, links and the average degree of the network^44^.

### Selection of the specific connector nodes

As explained in ref. 32, the centrality of the connector nodes linking independent networks may be crucial in the final distribution of centrality. The central nodes (C) of a network are those nodes with the largest eigenvector centrality, whereas the peripheral nodes (P) are those nodes with very low centrality (see Supplementary Note 2 for more details). When connecting two networks, the connector nodes allow to distinguish between central–central (CC) connections or peripheral–peripheral (PP) connections. Importantly, when a network-of-networks is broken into disconnected components, the cluster with the largest eigenvalue acquires all centrality, whereas the rest of the (weak) components accumulate zero centrality. PP connections lead to a scenario close to the disconnected case, thus pushing almost the totality of the centrality towards the strong network. Accordingly, any connection strategy based on PP links is practically equivalent to refusing to connect with any other network. For this reason, we have restricted the networks' strategies to CC connections or no connections (refusal to connect).

Finally, it is worth noting that the rules of the competition allow the networks to connect through more than one link (for example, in A↔B). For simplicity, throughout the examples studied in this work, we represent *w* connector links between networks as one link of weight *w*. However, in certain systems, links with weight larger than one could have no real meaning. In those cases, the second (third, etc.) link between two networks should be built between their second (third, etc.) most central nodes, maintaining the phenomenology qualitatively unchanged.

### The adjacency matrix and the dynamical processes

A variety of dynamical processes occurring in a network can be mathematically described as **n**(*t*+1)=**Mn**(*t*), where **n**(*t*) is a vector whose components are the state of each node at time *t* (for example, the population of individuals at each node), and **M**, with *M*_*ij*_≥0, is a matrix that contains the peculiarities of the dynamical process. As **M** is a primitive matrix, its largest eigenvalue is positive, it verifies that *λ*_1_>|*λ*_*i*_|, ∀ *i*>1 and its associated eigenvector is also positive. Therefore, the dynamics of the whole system is given by


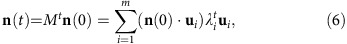


where **n**(0) is the initial condition and **u**_*i*_ the *i*th eigenvectors of **M**. From equation (6) we obtain that the system evolves towards an asymptotic state independent of the initial condition and proportional to the first eigenvector **u**_1_:


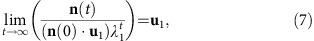


whereas its associated eigenvalue *λ*_1_ yields the growth rate at the asymptotic equilibrium. If **n**(*t*) is normalized such that |**n**(*t*)|=1 after each iteration, **n**(*t*)→**u**_1_ when *t*→∞ and there is a correspondence between the eigenvector centrality and the asymptotic state of the system at equilibrium: both the eigenvector centrality and the asymptotic state of the system are proportional to the eigenvector associated with the largest eigenvalue of the transition matrix **M**.

Regarding the phenomenology presented in this work, in case we were concerned about a specific dynamical process, we would replace the adjacency matrix **G** by the transition matrix **M**, obtaining the eigenvector centrality retained by each network with no loss of generality.

### Data availability

All relevant data are available from the authors upon request.

## Additional information

**How to cite this article:** Iranzo, J. *et al.* Competition among networks highlights the power of the weak. *Nat. Commun.* **7,** 13273 doi: 10.1038/ncomms13273 (2016).

**Publisher's note:** Springer Nature remains neutral with regard to jurisdictional claims in published maps and institutional affiliations.

## Supplementary Material

Supplementary Information Supplementary Figures 1-18, Supplementary Tables 1-3, Supplementary Notes 1-6 and Supplementary References.
