# Supplementary material for: Competition among networks highlights the power of the weak
Source: Nat Commun. 2016 Nov 14;7:13273. doi: 10.1038/ncomms13273 (PMC5114531; doi:10.1038/ncomms13273)
Supplement: Supplementary Information — Supplementary Figures 1-18, Supplementary Tables 1-3, Supplementary Notes 1-6 and Supplementary References. [file ncomms13273-s1.pdf]

SUPPLEMENTARY INFORMATION

Competition among networks highlights the unexpected power of the weak

J. Iranzo, J. M. Buldú and J. Aguirre

Supplementary Figures

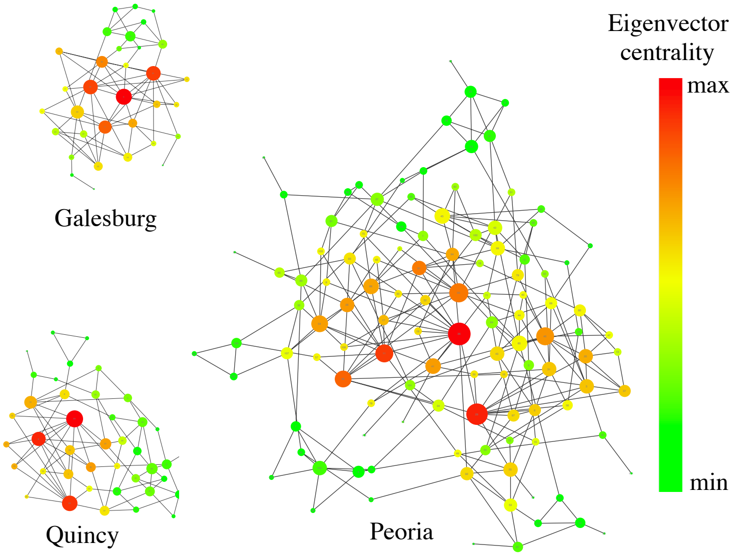

Supplementary Figure 1. Collaboration network among physicians in Peoria, Galesburg and Quincy (Illinois, US). Network parameters of each city are summarized in Supplementary Table 2. In the figure, the size of the nodes is proportional to the their number of connections, while colours reflect the eigenvector centrality of each node inside its city. Note that centralities are calculated before introducing connections between cities.

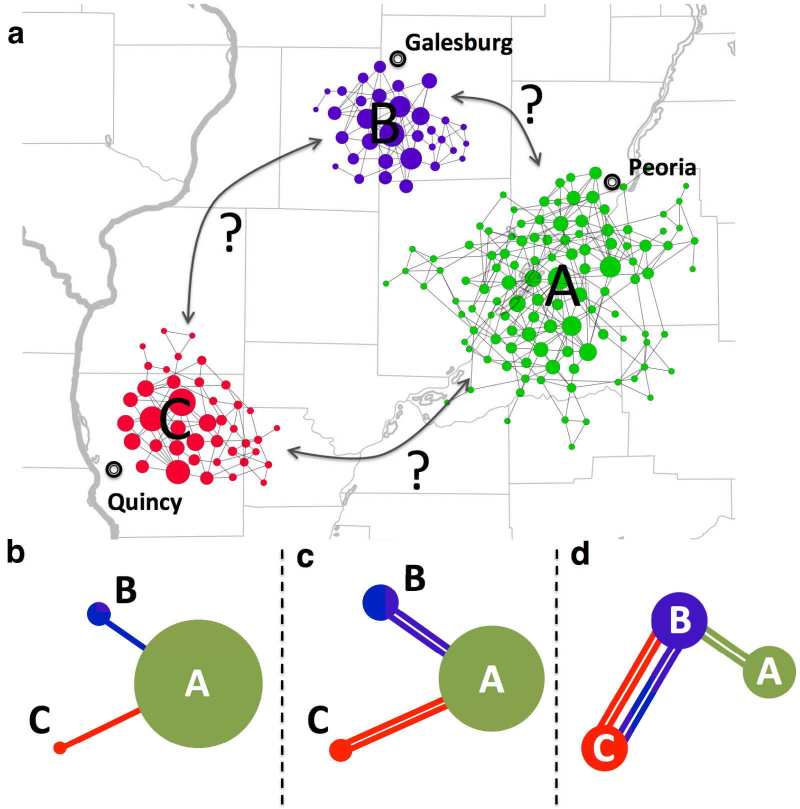

Supplementary Figure 2. Competition for centrality among professional networks. (a) Collaboration networks of physicians working in Peoria (A), Galesburg (B) and Quincy (C) (Illinois, US), from [10]. Creating connections between physicians leads to a network-of-networks T, whose centrality is distributed among the cities. In (b), (c) and (d) we show the centrality retained at each network depending on different connection strategies. The radius of each circle is proportional to the centrality accumulated by each network. Network strengths are $\lambda_A = 6.16$, $\lambda_B = 5.78$ and $\lambda_C = 5.39$. In (b), when one connection between physicians is allowed ($l = 1$), the unique Nash equilibrium leads to a centrality distribution of $C_A = 0.78$, $C_B = 0.14$ and $C_C = 0.08$. When networks are allowed to create two connections ($l = 2$), two solutions coexist: networks B and C obtain weak outcomes (c) when connecting to A (X'_∞: $C_A = 0.65$, $C_B = 0.21$ and $C_C = 0.14$), but their best strategy is to create the links between them, thus, forcing network A to join them (X'_0: $C_A = 0.33$, $C_B = 0.39$ and $C_C = 0.28$). In summary, the final result of the contest is largely dependent on the reached solution.

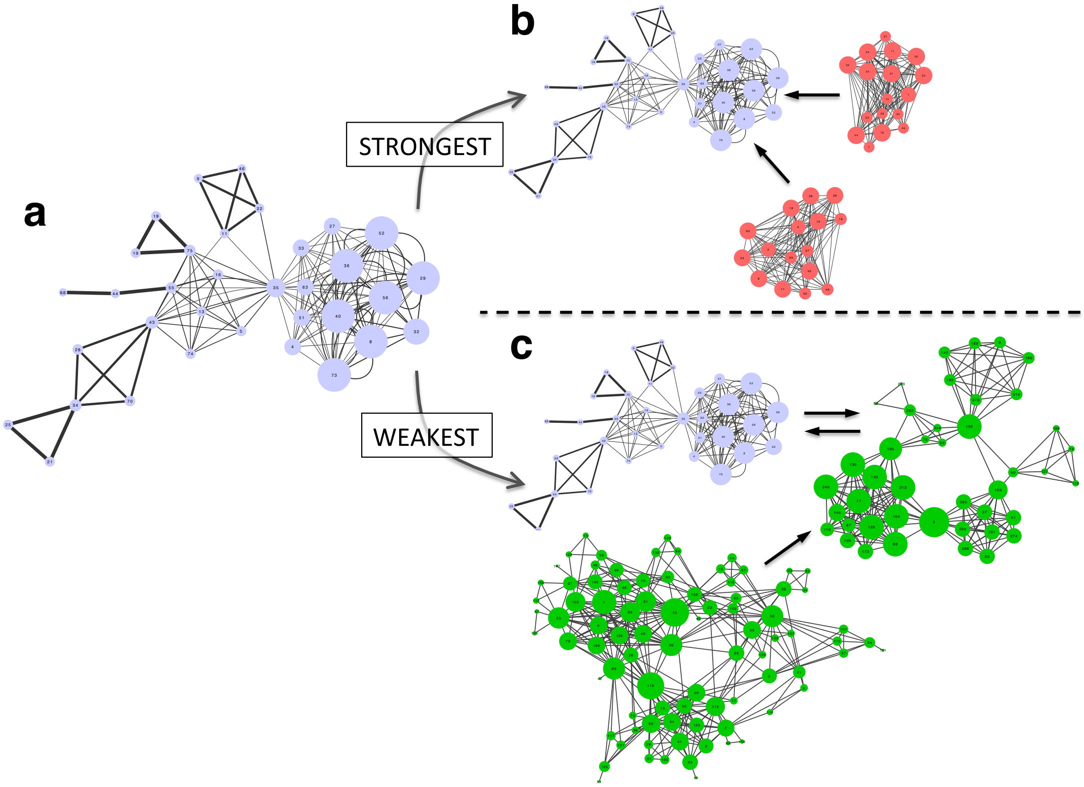

Supplementary Figure 3. Competition for centrality in scientific collaboration networks. (a) Giant component of the collaboration network of researchers of the University of Oxford who investigate on Ebola. The width of the links is proportional to their weights, which account for the strength of the collaboration between two authors based on the appearance as co-authors in the same paper (as explained in the text). Node size is proportional to the eigenvector centrality. Before connecting to other centres, Oxford needs to evaluate its strength compared to the other co-authorship networks, in order to choose the optimum strategy. (b) Oxford connects with other two centres whose collaboration networks are weaker, being X_∞ the most convenient Nash equilibrium. (c) Now, Oxford connects with centres with stronger collaboration networks and X_0 turns out to be its most convenient Nash equilibrium.

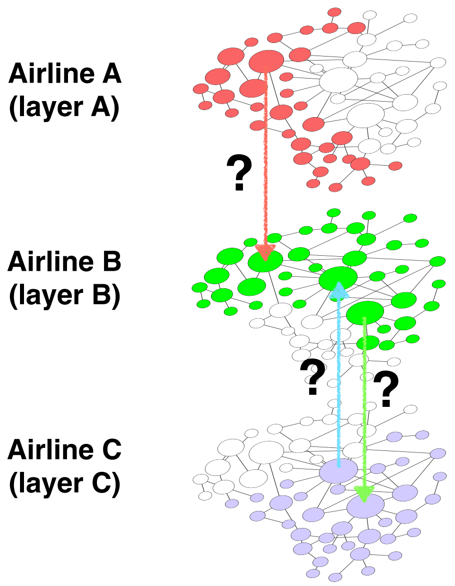

Supplementary Figure 4. Competition for centrality between commercial airlines. In this illustrative example, each network – i.e. layer – corresponds to a different commercial airline. The nodes of the networks represent airports, which are linked together if a flight (with a certain airline) between two airports exists. Colours red, green and blue indicate those airports where a given airline has flights, while uncoloured nodes are the airports not reached by the airlines. Two layers are connected when two airlines decide to make an agreement and connect flights at a given airport. In this case, each layer of the multi-layer network corresponds to a subnetwork that is competing for acquiring the highest possible centrality. Note that the adequate election of a partner airline can lead to a higher increase of the centrality of a given company (or a pair of them) in the global air transportation network.

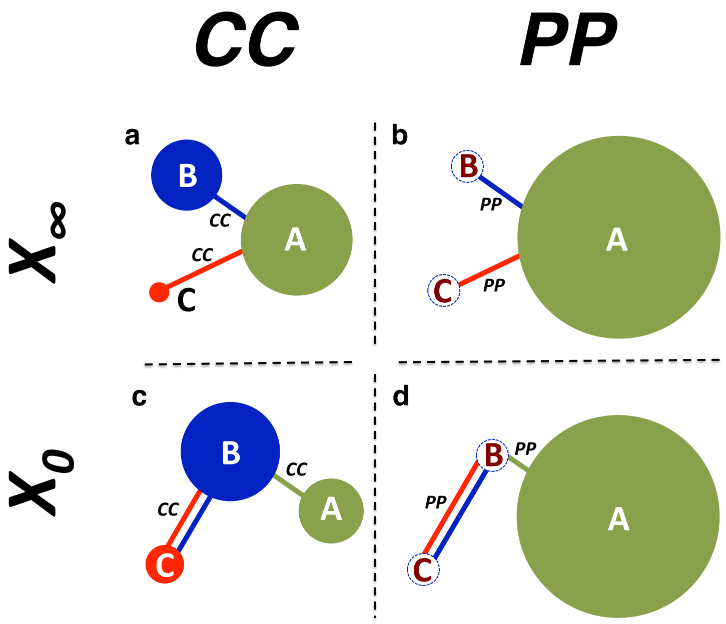

	$C(X_\infty)$ equilibrium		$C(X_0)$ equilibrium	
Strategy:	CC	PP	CC	PP
Network A	0.55	0.999999696	0.32	0.999999712
Network B	0.35	0.000000150	0.49	0.000000191
Network C	0.10	0.000000154	0.19	0.000000097

Supplementary Figure 5. Differences in the distribution of centrality for central-central (CC) and peripheral-peripheral (PP) connection strategies. The circles represent the loan networks of three independent Indian villages (see Fig. 1 of the main manuscript for details). Creating connections between villages would lead to a network-of-networks T, whose centrality is distributed among the villages. Each picture shows the centrality retained at each village (network) for (a) solution $X_\infty = \{A(0), B \to A, C \to A\}$ and CC connection, (b) solution X_∞ and PP connection, (c) solution $X_0 = \{A \to B, B \leftrightarrows C\}$ and CC connection, and (d) solution X_0 and PP connection. The radius of each circle is proportional to the centrality accumulated by each network, and circles of radius close to zero are plotted in dashed line (see the table for the actual values). Networks' strengths are $\lambda_A = 4.27$, $\lambda_B = 4.05$ and $\lambda_C = 3.38$.

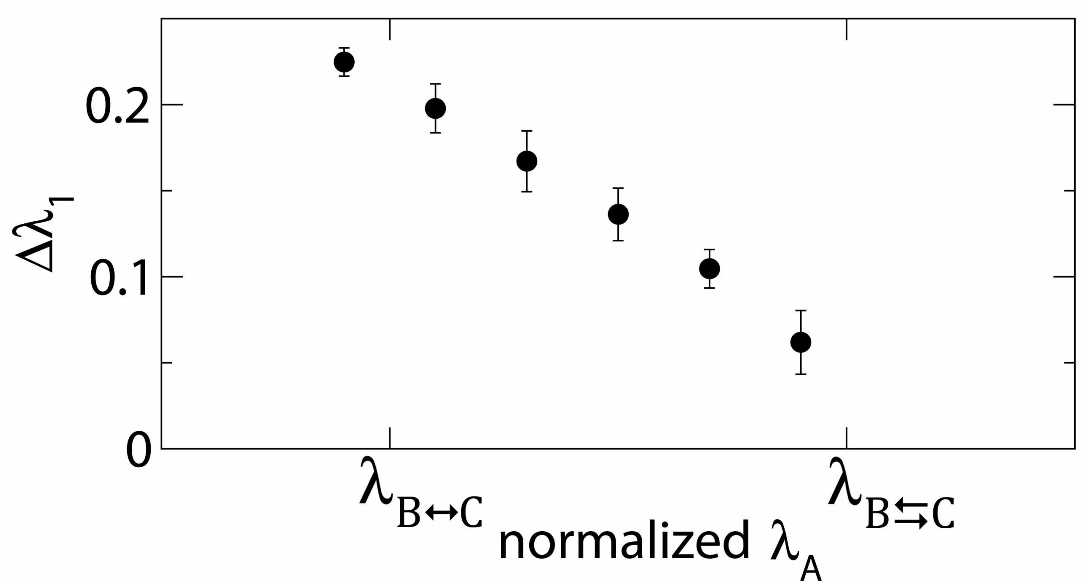

Supplementary Figure 6. Difference between the total strength of solutions X_0 and X_∞, $\Delta\lambda_1 = \lambda_1(X_0) - \lambda_1(X_\infty)$, as a function of the strength of the strong network in the competition for centrality among 3 networks. Data points (error bars) correspond to averages (standard deviations) over all realizations whose $\lambda_{A,1}$ lies in the corresponding X-axis interval. Note that the strength of solution X_0, the cooperative equilibrium, is always larger than the strength of solution X_∞, the equilibrium based on weak networks connecting to the strong one. The realizations are developed with the networks generated with the Barabási-Albert model [27] used in Fig. 2 of the main text: each competitor makes use of as much as $l = 1$ link to connect with the rest of the networks, for each choice of B and C the system is solved for 20 series of A, and results are an average of more than 500 sets of A, B, and C. The X-axis has been rescaled to allow for comparisons among different realizations.

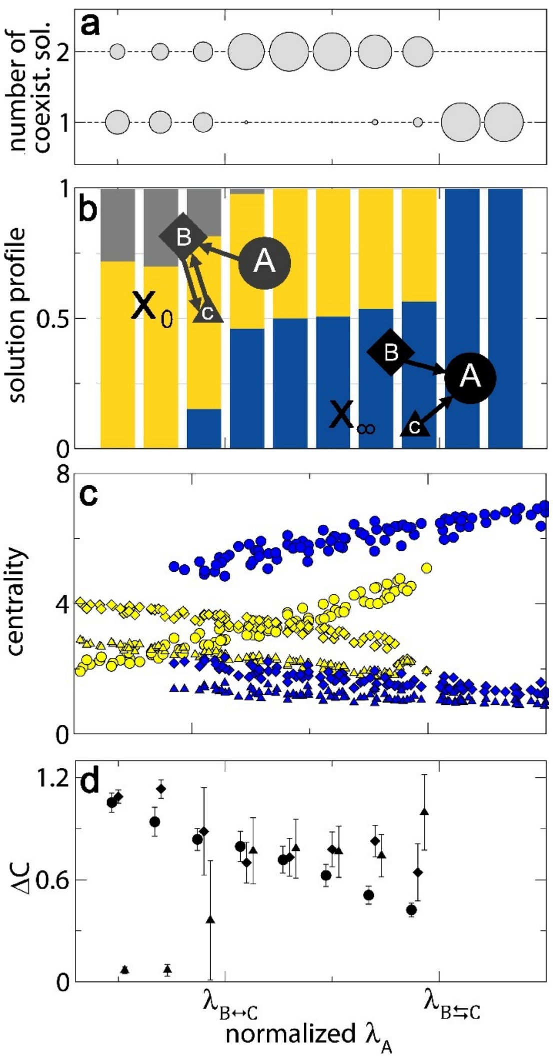

Supplementary Figure 7. Competition for centrality among 3 networks as a function of the strength of the strong network, when the centrality is defined as $\mathcal{C}'_i = \lambda_{T,1}\mathcal{C}_i$. Each competitor makes use of as much as $l = 1$ connector link. For each choice of weak networks B and C, the system is solved for 20 series of the strong network A, and results are an average of more than 500 sets of A, B, and C (see caption of Fig. 2 of main manuscript for more details). (a) Number of coexisting Nash equilibria per realization. The radius of each circle is proportional to the fraction of realizations. (b) Relative occurrence of different configurations in the set of solutions, averaged over all realizations: (i) Equilibrium $X_0 = \{A \to B,\, B \leftrightarrows C\}$ (yellow), (ii) equilibrium $X_\infty = \{A(0), B \to A, C \to A\}$ (blue) and (iii) other equilibria (grey). (c) Centrality of networks A ($\circ$), B ($\diamond$) and C ($\triangle$) for a particular choice of B and C ($\lambda_B = 5.25$ and $\lambda_C = 5.2$). The results are shown for solutions X_0 and X_∞ (colour code as in (b)). (d) Relative centrality variability ΔC among different Nash equilibria. Data points (error bars) correspond to averages (standard deviations) over all realizations whose λ_A lies in the corresponding X-axis interval. Network symbols as in (c).

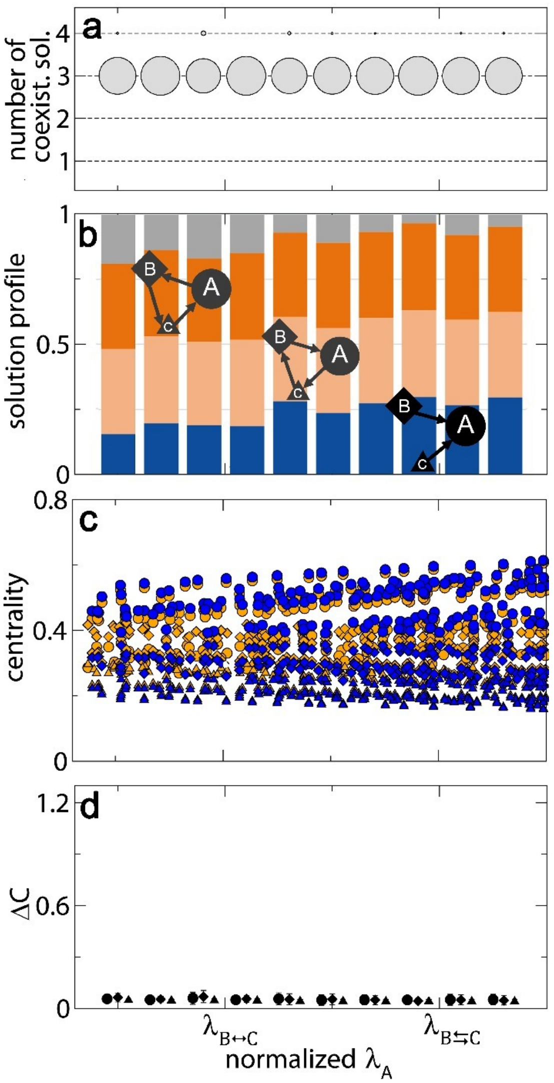

Supplementary Figure 8. Competition for betweenness centrality among 3 networks as a function of the strength of the strong network. Each competitor makes use of as much as $l = 1$ link to connect with the rest of the networks. For each choice of weak networks B and C, the system is solved for 20 series of the strong network A, and results are an average of more than 500 sets of A, B, and C (see caption of Fig. 2 of main manuscript for more details). The centrality of each network is measured as its betweenness centrality. (a) Number of coexisting Nash equilibria per realization. The radius of each circle is proportional to the fraction of realizations. (b) Relative occurrence of different configurations in the set of solutions, averaged over all realizations: (i) Equilibrium $X_1 = \{A \to B, B \to C, C \to A\}$ (dark orange), (ii) equilibrium $X_2 = \{A \to C, C \to B, B \to A\}$ (pale orange), (iii) $X_\infty = \{A(0), B \to A, C \to A\}$ (blue) and (iv) other equilibria (grey). (c) Centrality of networks A ($\circ$), B ($\diamond$) and C ($\triangle$) for a particular choice of B and C ($\lambda_{B,1} = 5.25$ and $\lambda_{C,1} = 5.2$). The results are shown for solutions X_0 (blue) and $X_1 + X_2$ (orange). (d) Relative centrality variability ΔC among different Nash equilibria. Data points (error bars) correspond to averages (standard deviations) over all realizations whose $\lambda_{A,1}$ lies in the corresponding X-axis interval. Network symbols as in (c). It is clear that the benefit for migrating from one solution to another is negligible.

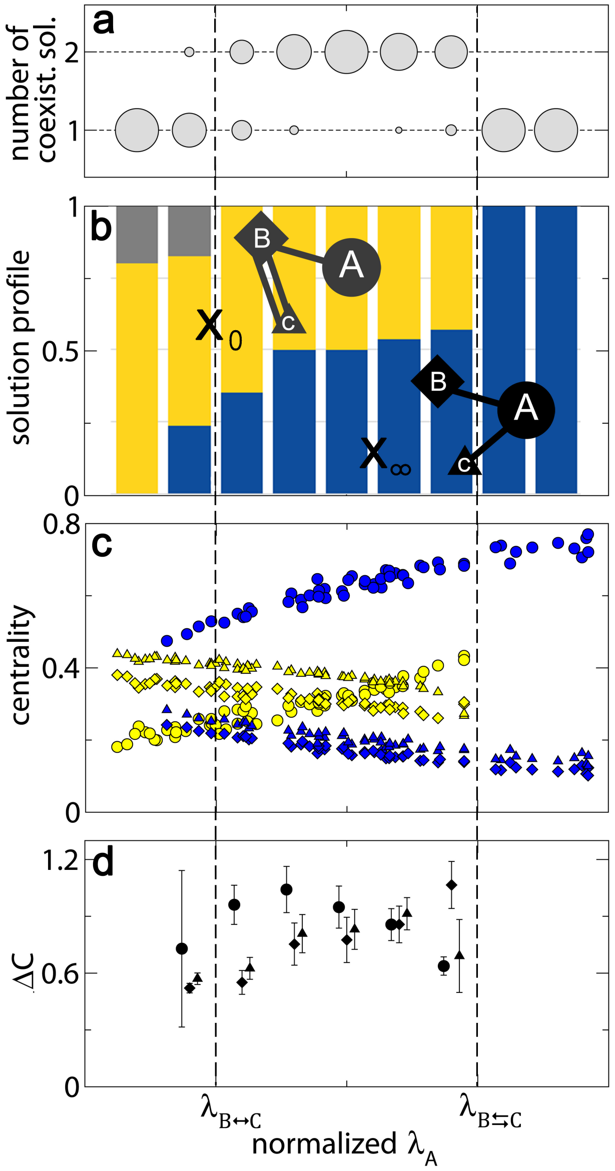

Supplementary Figure 9. Competition for centrality among 3 Erdős-Rényi networks as a function of the strength of the strong network. Each competitor makes use of as much as $l = 1$ link to connect with the rest of the networks. We modify the size and/or mean degree of network A in order to increase its strength from $\lambda_{A,1} = \lambda_{B,1}$ to $\lambda_{A,1} \gg \lambda_{B \leftrightarrows C,1}$. The X-axis has been rescaled to allow for comparisons among different realizations. (a) Number of coexisting Nash equilibria per realization. The radius of each circle is proportional to the fraction of realizations. (b) Relative occurrence of different configurations in the set of solutions, averaged over all realizations: (i) Equilibrium $X_0 = \{A \to B,\, B \leftrightarrows C\}$ (yellow), (ii) equilibrium $X_\infty = \{A(0), B \to A, C \to A\}$ (blue) and (iii) other equilibria (grey). (c) Centrality of networks A ($\circ$), B ($\diamond$) and C ($\triangle$) for a particular choice of B and C ($\lambda_{B,1} = 5.25$ and $\lambda_{C,1} = 5.2$). The results are shown for solutions X_0 and X_∞ (colour code as in (b)). (d) Relative centrality variability ΔC among different Nash equilibria. Data points (error bars) correspond to averages (standard deviations) over all realizations whose $\lambda_{A,1}$ lies in the corresponding X-axis interval. Network symbols as in (c).

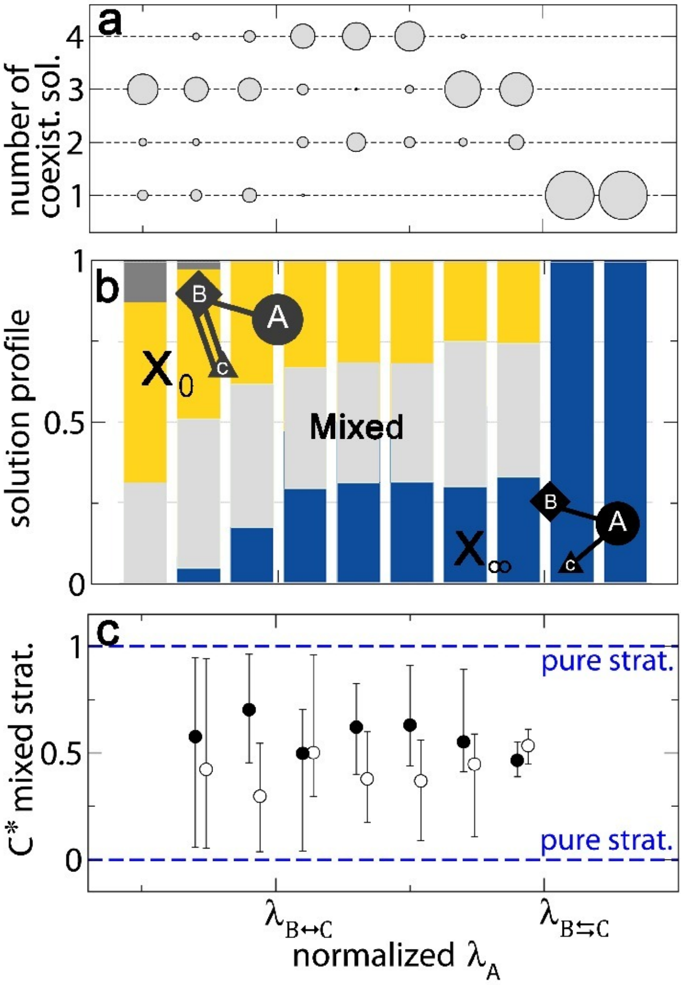

Supplementary Figure 10. Competition for centrality among 3 networks as a function of the strength of the strong network, when both pure and mixed strategies are allowed. In this example $l = 1$, which means that each network can connect through weighted connector links to all its competitors as far as the addition of such weights is equal to or less than 1. For each choice of weak networks B and C, the system is solved for 20 series of the strong network A, and results are an average of more than 500 sets of A, B, and C (see caption of Fig. 2 of main manuscript for more details). (a) Number of coexisting Nash equilibria per realization. The radius of each circle is proportional to the fraction of realizations (no cases with more than four coexisting solutions were found). (b) Relative occurrence of different configurations in the set of solutions, averaged over all realizations: (i) Pure equilibrium $X_0 = \{A \to B,\, B \leftrightarrows C\}$ (yellow), (ii) pure equilibrium $X_\infty = \{A(0), B \to A, C \to A\}$ (blue), (iii) other pure equilibria (dark grey), and (iv) mixed equilibria (pale grey). Note that (a) and (b) correspond to Figs. 2a and 2b, but where mixed equilibria are allowed. (c) Mixed-vs-pure relative centrality, C^*. Here, $C^* = 1$ and $C^* = 0$ correspond to the centralities of a network under its most and least desirable pure equilibria, respectively. The plot shows the mean values of C^* for the strong network (•), the coalition of the small networks (○), and the 5/95 percentiles (error bars).

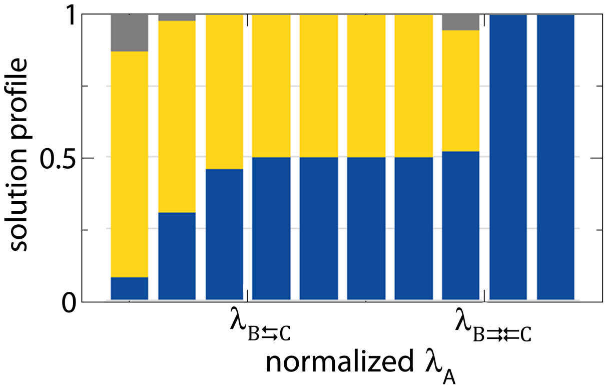

Supplementary Figure 11. Solution profiles for a competition in which each network can create up to two connector links, as a function of the strength of the strong network. Each competitor makes use of as much as $l = 2$ links to connect with the rest of the networks. For each choice of weak networks B and C, the system is solved for 20 series of the strong network A, and results are an average of more than 500 sets of A, B, and C. Bars indicate the relative occurrence of different configurations in the set of solutions, averaged over all realizations, as a function of the strength of A. Solutions are classified in three groups (yellow: equilibrium $X_0' = \{A \rightrightarrows B, B \rightrightarrows\leftleftarrows C\}$, blue: equilibrium $X_\infty' = \{A(0), B \rightrightarrows A, C \rightrightarrows A\}$, grey: other equilibria).

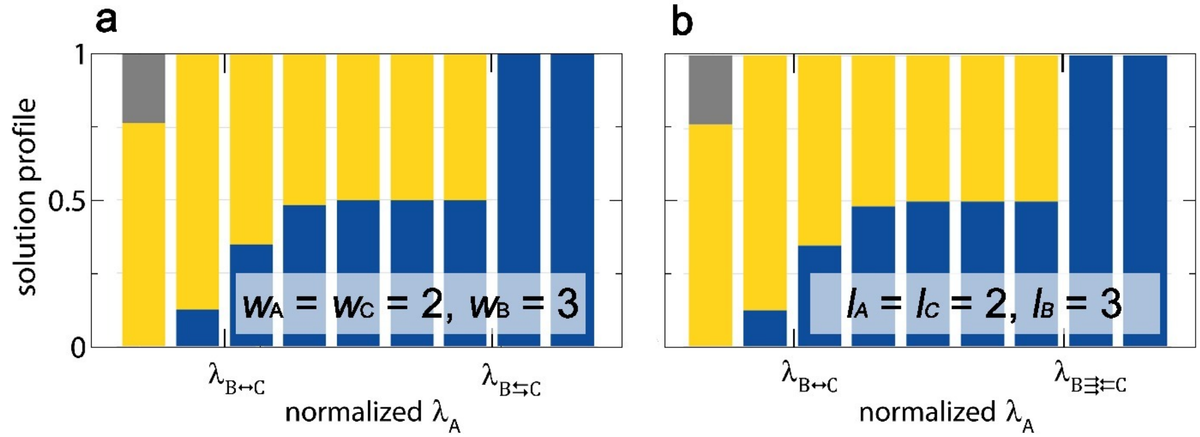

Supplementary Figure 12. Comparison between the solution profiles for a competition among 3 networks relative to the cases where each network has different maximum number of connector links and different weights in the connector links. For each choice of weak networks B and C, the system is solved for 20 series of the strong network A, and results are an average of more than 500 sets of A, B, and C. Bars indicate the relative occurrence of different configurations in the set of solutions, averaged over all realizations, as a function of the strength of A. Two different cases are studied: (a) Different maximum number of connector links: $l_A = l_C = 2$ and $l_B = 3$, with $w_A = w_B = w_C = 1$. (b) Different weights in the connector links: $w_A = w_C = 2$ and $w_B = 3$, with $l_A = l_B = l_C = 1$. Solutions are classified in three groups (yellow: equilibrium $X_0 = \{A \rightarrow B,\ B \leftrightarrows C\}$, blue: equilibrium $X_\infty = \{A(0), B \rightarrow A, C \rightarrow A\}$, grey: other equilibria). Note that both plots are identical.

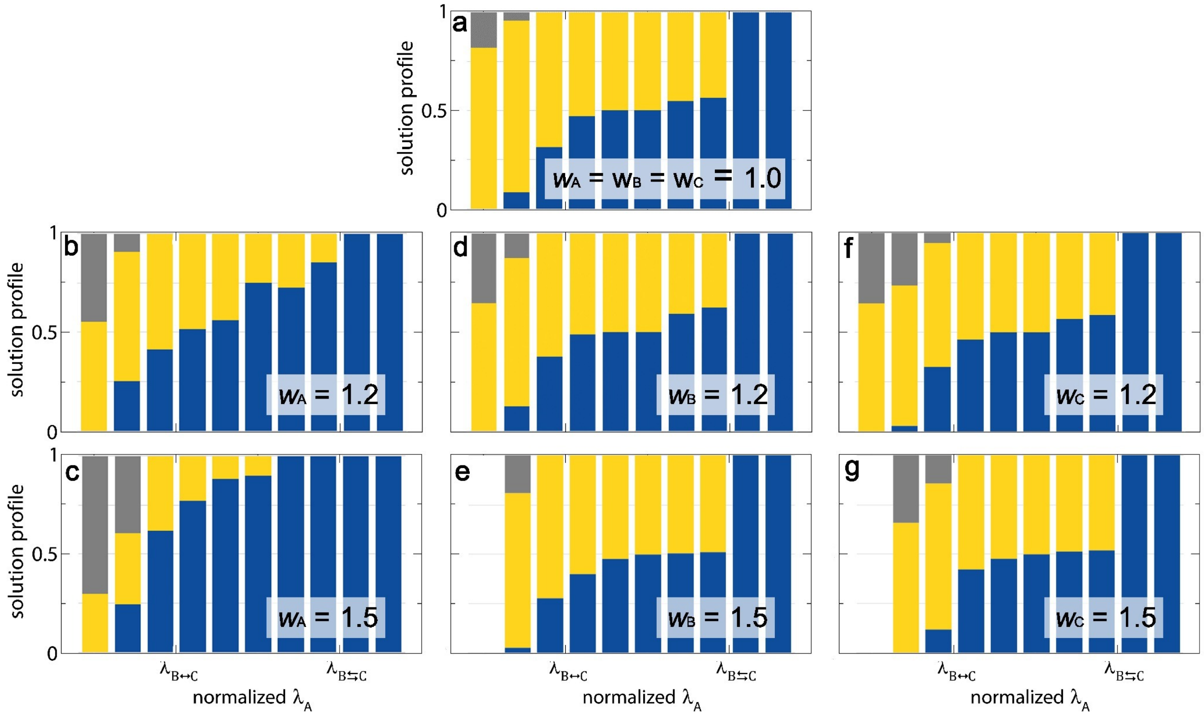

Supplementary Figure 13. Solution profiles for a competition among 3 networks where one network increases the weight of their connector links, as a function of the strength of the strong network. Each competitor makes use of as much as $l = 1$ link to connect with the rest of the networks. For each choice of weak networks B and C, the system is solved for 20 series of the strong network A, and results are an average of more than 500 sets of A, B, and C. Bars indicate the relative occurrence of different configurations in the set of solutions, averaged over all realizations, as a function of the strength of A. (a) $w_A = w_B = w_C = 1$ (equivalent to Fig. 2 in the main text). Increase in w_A: (b) $w_A = 1.2, w_B = w_C = 1$ and (c) $w_A = 1.5, w_B = w_C = 1$. Increase in w_B: (d) $w_A = w_C = 1, w_B = 1.2$ and (e) $w_A = w_C = 1, w_B = 1.5$. Increase in w_C: (f) $w_A = w_B = 1, w_C = 1.2$ and (g) $w_A = w_B = 1, w_C = 1.5$. Solutions are classified in three groups (yellow: equilibrium $X_0 = \{A \to B,\, B \leftrightarrows C\}$, blue: equilibrium $X_\infty = \{A(0), B \to A, C \to A\}$, grey: other equilibria).

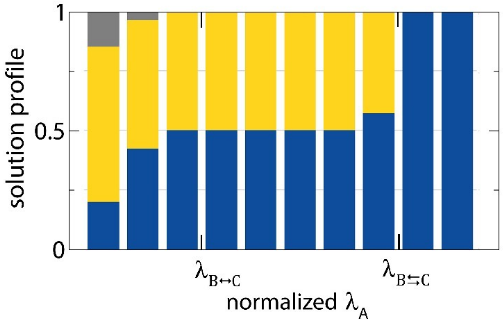

Supplementary Figure 14. Solution profiles for a competition among 3 networks A, B, and C of size 10 nodes as a function of the strength of the strong network. Each competitor makes use of as much as $l = 1$ link to connect with the rest of the networks. Results are an average of 100 sets of A, B, and C. Bars indicate the relative occurrence of different configurations in the set of solutions, averaged over all realizations, as a function of the strength of A. Bars indicate the relative occurrence of different configurations in the set of solutions as a function of the strength of A. Solutions are classified in three groups (yellow: equilibrium $X_0 = \{A \to B,\ B \leftrightarrows C\}$, blue: equilibrium $X_\infty = \{A(0), B \to A, C \to A\}$, grey: other equilibria).

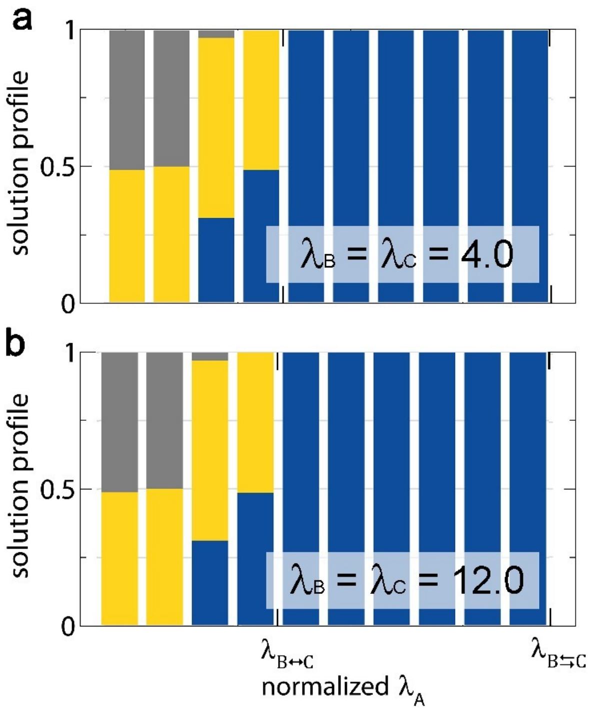

Supplementary Figure 15. Solution profiles for a competition among 3 networks A, B, and C of size 1 node as a function of the strength of the strong network. The strength of each 1 node-network $\lambda_{i,1}$ is equal to the weight of the auto-loop associated with each node. Bars indicate the relative occurrence of different configurations in the set of solutions as a function of the strength of A. Solutions are classified in three groups (yellow: equilibrium $X_0 = \{A \to B,\ B \leftrightarrows C\}$, blue: equilibrium $X_\infty = \{A(0), B \to A, C \to A\}$, grey: other equilibria).

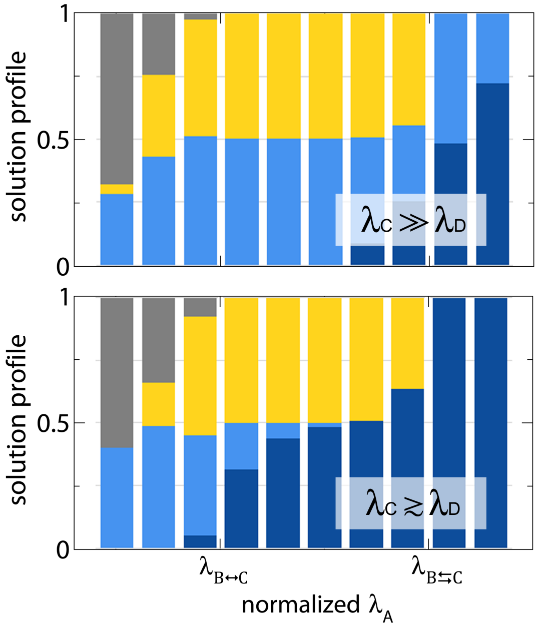

Supplementary Figure 16. Solution profiles for a competition among 4 networks A, B, C and D as a function of the strength of the strong network. $\lambda_{A,1} > \lambda_{B,1} > \lambda_{C,1} > \lambda_{D,1}$. Each competitor makes use of as much as $l = 1$ link to connect with the rest of the networks. For each choice of weak networks B and C, the system is solved for 20 series of the strong network A, and results are an average of more than 500 sets of A, B, and C. Results are shown for two possible sizes of the weakest network D: much smaller than C ($\lambda_{D,1} = 0.6\lambda_{C,1}$, upper panel) and similar to C ($\lambda_{D,1} = 0.9\lambda_{C,1}$, lower panel). Bars indicate the relative occurrence of different configurations in the set of solutions according to the following classification: Yellow: equilibrium $X_0'' = \{A \to B, B \leftrightarrows C, D \to B\}$; dark blue: equilibrium $X_\infty'' = \{A(0), B \to A, C \to A, D \to A\}$; light blue: equilibrium $X_{\infty,P}'' = \{A \leftrightarrows D, B \to A, C \to A\}$; grey: other equilibria. Note that equilibria X_∞'' and $X_{\infty,P}''$ cannot appear together in the same realization and the latter can be considered a perturbation of the former.

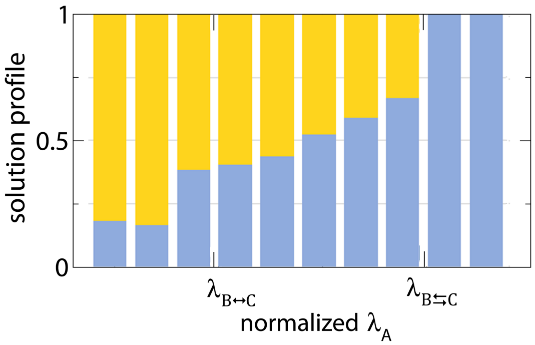

Supplementary Figure 17. Competition for centrality among 3 scale-free networks as a function of the strength of the strong network when the payoff is that of the central nodes. Each competitor makes use of $l = 1$ connector link. For each choice of weak networks B and C, the system is solved for 20 series of the strong network A, and results are an average of more than 500 sets of A, B, and C (see caption of Fig. 2 of main manuscript for more details). Relative occurrence of different configurations in the set of solutions, averaged over all realizations, when the payoff is the connector nodes' centrality divided by the addition of the centralities of the rest of nodes: (i) Equilibrium $X_0 = \{A \to B,\ B \leftrightarrows C\}$ (yellow), and (ii) equilibrium $X_\infty' = \{A \to C, B \to A, C \to A\}$ (light blue).

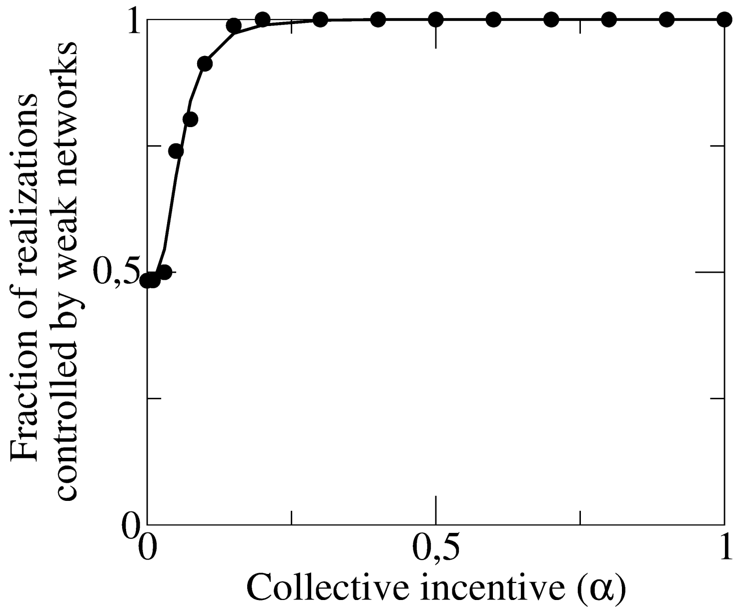

Supplementary Figure 18. Cooperation and control of the game by weak networks (*power of the weak*) when the payoff is a combination of the collective centrality and the individual centrality of connector nodes. Each competitor makes use of $l = 1$ connector link. For each choice of weak networks B and C, the system is solved for 20 series of the strong network A, and results represent fractions over more than 100 sets of A, B, and C scale-free (Barabási-Albert) networks [27] (black), such that $\lambda_{B \leftrightarrow C} < \lambda_A < \lambda_{B \leftrightarrows C}$ (see caption of Fig. 2 of main manuscript for more details). The Y-axis represents the fraction of realizations in which (i) weak networks share the same preferred equilibrium, (ii) such equilibrium involves cooperation between weak networks, and (iii) weak networks can impose their preferred equilibrium on the strong network (migrations towards cooperation are possible). $\alpha = 0$ is the situation in which the payoff is that of the connector node individually, and $\alpha = 1$ is the case in which the payoff is that of the whole network.

Supplementary Tables

Network	N	L	λ_1	$\langle k \rangle$	C	d	k_{max}	$\max\{u_1\}$
Village A	148	193	4.267	2.61	0.115	7.37	8	0.491
Village B	56	66	4.054	2.36	0.021	5.08	10	0.529
Village C	43	47	3.380	2.19	0.094	6.16	7	0.398

Supplementary Table 1. Summary of the main parameters of the loan networks of villages A, B and C (in [1], villages 55, 15 and 14, respectively). Specifically, the parameters shown in the table are: size of the networks N, number of connections within each village L, largest eigenvalue of the connectivity matrix λ_1, average degree (i.e., number of connections) of the network $\langle k \rangle$, clustering coefficient C, average shortest path of the network d, highest degree k_{max} and eigenvector centrality of the most central node $\max\{u_1\}$.

Network/City	N	L	λ_1	$\langle k \rangle$	C	d	hub	k_{max}	$\max\{u_1\}$
Peoria	113	246	6.157	4.354	0.216	3.606	36	14	0.33
Galesburg	33	71	5.776	4.303	0.329	2.669	33	10	0.412
Quincy	41	82	5.387	4.0	0.254	2.985	24	10	0.433

Supplementary Table 2. Summary of the main parameters of the collaboration networks of Peoria, Galesburg and Quincy [10]. Specifically, the parameters shown in the table are: size of the networks N, number of connections within each city L, highest eigenvalue of the connectivity matrix λ_1, average degree (i.e., number of connections) of the network $\langle k \rangle$, clustering coefficient C, average shortest path of the network d, node with the highest degree (hub), highest degree k_{max} and eigenvector centrality of the most central node $\max\{u_1\}$.

(A) Oxford co-authorship network being the strongest					
Institution	N	L	λ_1	$C(X_\infty)$	$C(X_0)$
Oxford	34	159	1.273	0.535	0.255
Network B	17	115	0.892	0.276	0.446
Network C	16	112	0.674	0.184	0.299
(B) Oxford co-authorship network being the weakest					
Institution	N	L	λ_1	$C(X_\infty)$	$C(X_0)$
Network A	86	346	2.106	0.757	0.406
Network B	43	190	1.437	0.148	0.370
Oxford	34	159	1.273	0.095	0.224

Supplementary Table 3. Summary of the main parameters of the Oxford co-authorship network before and after connecting to other two research centres. The competing networks have been generated with the model described in the text in order to fulfil that: (A) Oxford is the strongest network and (B) Oxford is the weakest network. $C(X_\infty)$ accounts for the Nash equilibrium where the two weak networks attach to the strongest one, while $C(X_0)$ is the Nash equilibrium where the two weaker network are linked together, forcing the strong one to connect to them. The parameters shown in the table are: size of the networks N, number of connections within each research centre L, highest eigenvalue of the connectivity matrix λ_1. The last two columns account for the total centrality accumulated for each network in the $C(X_\infty)$ and $C(X_0)$ equilibria.

Supplementary Note 1. Nash equilibria in network-of-networks: Applications to real cases

In this Supplementary Note we overview a series of cases in which the methodology introduced in the main text could be applied. Importantly, all these examples, where networks with a certain identity interact creating a network-of-networks, must accomplish two fundamental requirements:

- Every node should belong to a single network or community, that is, networks must not overlap.
- All the nodes of a network must behave as a whole competing entity. In practice, this means that the strategies followed by each network during competition pursue the improvement of the whole network importance, not that of certain individuals. This requirement can be fulfilled either by (i) all members of the community (i.e., nodes of the network) sharing a common objective, or (ii) by the existence of a single agent controlling the strategies of the network.

Once these two constraints are satisfied, the methodology proposed here can be applied, regardless of the underlying system. Thus, a diversity of social, technological and biological networks can be inspected under the proposed framework. Certainly, social networks with some degree of hierarchy are the most suitable candidates, since their modular structure combined with the existence of agents that promote coordinated actions (local authorities, funding agencies, professional associations) make these two constraints easily fulfilled. As we will see, loan networks, scientific collaboration networks or physician networks are good examples. In addition, two possible applications to technological and biological systems will be discussed.

Social networks

◦ Loan networks in Indian villages

Loan networks are a good example of how our methodology could improve our understanding of the dynamics of interaction among networks. In the year 2006, a series of surveys were carried out at 75 villages in rural southern Karnataka, a state in the south of India [1]. The objective of these surveys was to understand the social interactions among inhabitants within each village, with the aim of carrying out a microfinance program consisting of granting small credits to develop the economy of the villages. Besides their economical interest, the surveys reported very relevant information about the social behaviour of the inhabitants of the villages, how they interacted with each other and which the main actors of such relations were.

In our study, we construct *loan networks* making use of the answers to the questions *"who would you lend money to?"* and *'from whom would you borrow money?"* of such surveys. As in [1], when the answer to both questions links two inhabitants of the village, we create a connection between them, leading to a binary (0/1) connectivity matrix $\mathbf{G}$. In turn, we consider the matrix to be symmetric, since when a loan is granted, money moves in both directions of the link –as sooner or later it will have to be paid back–. From the whole dataset of 75 villages, we selected three of them (from now on, villages A, B and C) and used them as an example to analyse how the connections between villages would affect the resulting *loan network-of-networks*. Details about the size, i.e. number of people (nodes) and interactions between them (links) can be found in Supplementary Table 1. Villages were named according to the value of the largest eigenvalue of their adjacency matrix, such that $\lambda_{1,A} > \lambda_{1,B} > \lambda_{1,C}$. As the strength of each network (village) is given by its largest eigenvalue, network A is the strongest and network C the weakest competitor.

Before investigating the consequences of connecting networks, we identified which are the most relevant nodes inside each village by means of the eigenvector centrality [2] (see Fig. 1a of the main manuscript).

Local loan networks constructed as explained above are based on *real* confidence among inhabitants of a community, and for this reason provide much information about some critical financial properties associated to such community [3, 4]. In particular, they cast light on the financial resilience of a region, that is, its capacity to overcome financial difficulties and systemic risks [5–7]. In our example, imagine the usual case of several close villages that base their economy on different products (e.g., one village grows mainly tobacco while the neighbours raise sugar cane, the two leading crops in Karnataka –see [8] for a detailed explanation on the agricultural techniques and challenges of such region–). If a plague or disease got into the crops and caused a lot of damage, the most affected village would benefit critically from the connections to other villages that would allow them to obtain (i) an alternative to that crop to ensure the feeding of its population and animals, and (ii) a way to obtain an effective financial backing to overcome the crisis. On the other hand, the rest of villages would profit from finding a new client for their products and from offering their financial assistance. While this is just a simple example, it clarifies that increasing the loan network of a village or region by promoting connections to other regions is doubtlessly an attractive strategy to successfully face unexpected climatic, natural or financial challenges.

How would the local authorities promote such connections to other villages? This fact will critically depend on the cultural, economical and social properties of the regions to connect. In developed regions useful ways are improving transportation and communications, or in case the nodes represented companies, by regional Chambers of Commerce promoting the connections between different businesses, through bilateral investment plans, etc. However, in our Indian case, the authorities would surely focus on promoting the social contact between villages –as loan networks are based on personal confidence–. To achieve this objective, in this context the local administrations frequently organize inter-regional social activities, such as feasts or festivals, that promote friendship and marriages between people of different villages. In particular, the connection between two especially wealthy families –the central nodes in our model–, would be very favourable for the economy of both villages.

Specifically, we can investigate in our particular example which village would accumulate more centrality/importance in the loan network, thus having a comparative advantage when compared to their interacting partners. As explained in the main text (see Fig. 1 and related text), once the three villages have been allowed to play the game of connecting to other villages, the centrality accumulated by each network strongly depends on the new connections. This way, villages B and C can overcome the importance of village A by creating connections between them. What is more, village A should not remain disconnected from B and C, since in the long run, it would lose centrality compared to the case of linking to the cluster formed by the two weakest networks. Furthermore, any of the weaker villages can lead the strategies in the connection process, snatching the strong village from the control of the whole network-of-networks. Finally, note that similar qualitative results would have been obtained with many other combinations of the 75 villages.

◦ Professional collaboration networks: The case of Illinois physicians

A similar example can be found in the context of professional collaboration networks. In such networks, nodes are individuals working in a specific field and the links of the network account for collaborations among them. The study of collaborations among American physicians in the middle fifties carried out by Coleman et al. [9, 10] is a seminal example of the analysis of this kind of networks. In that work, authors were concerned about the spreading of innovation along social networks. Between November 1953 and February 1955 a series of surveys were carried out in different cities of Illinois, with the aim of obtaining how the professional interactions between physicians led to the adoption of a new treatment.

We focused on the part of the Coleman's datasets that evaluated the collaboration network among physicians. Specifically, we analysed the answers to the question *"who are the three or four physicians with whom you most often find yourself discussing cases or therapy in the course of an ordinary week – last week for instance?"* [9]. The procedure was simple: each answer creates a bidirectional link between two physicians. This way, we obtained the collaboration networks of 3 different cities: Peoria, Galesburg and Quincy (see Supplementary Fig. 1). In all cases, cities were disconnected from each other, i.e., none of the medical doctors had weekly connections with counterparts in any of the other two cities.

As in the example of the Indian villages, the importance of the individuals belonging to each physician network was quantified by means of the eigenvector centrality: Supplementary Fig. 1 shows a qualitative description of such collaboration networks, with the node size proportional to the eigenvector centrality of the nodes.

The main parameters of the collaboration network of each city are summarized in Supplementary Table 2.

At this point we can address the consequences of connecting two cities. Let us suppose that a city – through its local Medical Council, for example– would like to promote the collaboration of their physicians with physicians of other cities (by means of a funding program, for example), with the aim of increasing their own knowledge and leadership in a certain field. What connections should the city promote? Note that if each network represented a hospital instead of a city, this model would simulate quite precisely the interconnection between hospitals through *fellowship* programs, a very established practice in some (mostly European) countries. Many hospitals typically finance the temporal stay of their medical doctors in other hospitals, with the aim of acquiring experience in a new medical or surgical technique. Once the stay is finished –usually lasts between one and two years–, the visitors go back to their original department and transmit the new technique to their colleagues, improving the *know-how* of the whole group and the capacity of the hospital of attracting patients.

In this way, if two physicians from different cities started to work together, the two collaboration networks would connect forming a network-of-networks and the importance of each city (i.e. network) could be measured by the importance accumulated by its physicians. When establishing these connections, could the cities *compete for importance*, thus promoting specific collaborations to increase their outcome? Which city would benefit the most from the knowledge exchanged by the *connecting* physicians?

As explained in the main text, the key metric to understand how the centrality is redistributed after networks creating new connections is the largest eigenvalue of the connectivity matrix λ_1. In turn, it allows to rank the strength of the cities leading to $\lambda_{Peoria} > \lambda_{Galesburg} > \lambda_{Quincy}$. Therefore, Peoria would be network A (the strongest), Galesburg would be network B and Quincy would be network C (the weakest).

Supplementary Figures 2a-d show the competition for centrality between these three cities. Allowing a single connector link ($l = 1$) between them, only one Nash equilibrium is obtained $\{A(0), B \to A, C \to A\}$, and the strongest network drastically outperforms its competitors (Supplementary Fig. 2b), i.e. Peoria (A) would strongly benefit from the interaction with physicians from any other city. However, if two interactions between physicians are allowed ($l = 2$), two coexisting solutions appear (see Supplementary Note 5 for more details on competitions where more than one connector link is accepted). Now, connecting to the strong city (solution $X'_\infty = \{A(0), B \rightrightarrows A, C \rightrightarrows A\}$, Supplementary Fig. 2c) leads to much worse

results for B and C than connecting with each other ($X_0' = \{A \rightrightarrows B, B \rightrightarrows\leftleftarrows C\}$, Supplementary Fig. 2d). In the latter case, the strong city must, in turn, connect to B to retain at least a small part of the centrality of the whole system, thus depending completely on the strategy adopted by the weak cities. And remind that any weak network can control the migration towards more beneficial equilibria (from X'_∞ to solution X_0'), while the strong network lacks the capacity to do the opposite.

○ Scientific collaboration networks: Investigating Ebola

Collaboration networks can also be studied in the framework of scientific co-authorship [11, 12]. In this case, the nodes of the network stand for researchers, who are linked when appearing together as co-authors of the same scientific publication [12]. Once again, for determining the importance of scientists from the co-authorship network we can use the eigenvector centrality, since it has become a traditional metric for quantifying the importance of a person inside a social network [13, 14]. Nevertheless, as explained above, we need to identify (or define) clusters or communities of researchers that, somehow, work with a common goal or, at least, belong to a community of some sort. For example, we can think of a research institute interested in improving its importance and its scientific production. Reasonably, the committee of the institute could pursue this goal by promoting the career of its scientists and establishing a collaboration visiting program. This way, encouraging professional connections to other centres in a way that the increase of centrality/importance of the research institute as a whole is larger, would be an interesting strategy for the institute's decision-makers. Under this framework, research centres –represented by the networks of their scientists' internal collaborations– would promote collaborations with other centres to generate a network-of-networks.

As an illustrative example, we selected the co-authorship network of those scientists who investigate Ebola at the University of Oxford –one of the world leading centres in the subject–. Specifically, we retrieved all published papers that appear at the Web of Science [15] containing the word Ebola in either the title or the abstract. Next, we selected only the connections between scientists belonging to the University of Oxford who have remained active during the last five years (period 2011-2015). If authors i and j participated in a paper of n_p authors, the weight of their connection would be $w_{ij} = 1/(n_p - 1)$, since the time devoted to collaborate is finite [11]. Additionally, the more papers authors i and j wrote together, the more weight was added to their link (following the same expression). In this way, we obtained the Oxford co-authorship network, which has a giant component of $N = 34$ scientists (see Supplementary Fig. 3 for details). Importantly, despite the links of this network are weighted, the methodology defined in the main text also applies.

Once a network of this type is constructed we can address how to evaluate the efficiency of tentative Collaboration Programs organized by the University of Oxford. Suppose that a certain amount of grants is given by the institution to promote collaborations with other research centres (e.g., covering travel expenses). When creating new links to other institutions, how would the University maximize its centrality compared to the others? Should the centre favour connections to weaker institutions or to stronger ones? To investigate these issues, an analysis of the distribution of centrality and the corresponding Nash equilibria would lead to helpful information for the Exchange Program Coordinators of the University.

Supplementary Figure 3 shows the Ebola co-authorship network of the University of Oxford, where node sizes and link widths are proportional to the eigenvector centralities and weights, respectively. Let us test now, by means of a simple model, how the creation of links with other institutions would modify the centrality of the Oxford co-authorship network. Note that, for simplicity, the link properties are based only on the number of published papers, but a much finer network could be built taking into account the impact factor of papers, their number of citations, etc. This would lead to an increase in the applicability and precision of the results.

To obtain the most productive connection strategies, we constructed a series of artificial co-authorship networks of different sizes. All of them were created with a model that generates papers with a random distribution of authors and links them with a weight inversely proportional to the number of authors (as

explained above). In this way, we obtained networks of different size N, number of links L and strength λ_1, which have structures similar to those of real co-authorship networks. Note that if we want to use networks associated to real research centres, these would be obtained following the steps explained above to create the network associated to the University of Oxford.

Importantly, when evaluating the consequences of connecting to other centres, the first issue is to determine whether the Oxford network is strong or weak in the group of potential competitors. Suppose that Oxford has the opportunity to collaborate with 2 other institutions whose co-authorship networks are weaker (i.e., $\lambda_{Oxford} > \lambda_B > \lambda_C$). Our methodology identifies which are the possible Nash equilibria of this situation and allows to quantify the gain of the University of Oxford in the collaboration network. Similarly, the same study can be carried out by considering the University of Oxford as the weakest network (i.e., $\lambda_A > \lambda_B > \lambda_{Oxford}$), allowing to assess how to connect to other weak networks to overcome a strong one.

Supplementary Table 3 summarizes the outcome, for the existing Nash equilibria, of the Oxford co-authorship network competing against two other test networks under two alternative scenarios (i.e., being the strongest and the weakest). As we can see, for the particular networks shown in Supplementary Table 3, the most productive strategy is to let weaker networks connect to Oxford and reach the X_∞ solution. However, according to the results presented in this manuscript, any of the weak networks could be able to push the whole system towards X_0 solution, and the centrality of the University of Oxford would decrease from 0.535 to 0.255. In that sense, the Centre Committee should decide whether they prefer this situation or the connection to stronger networks (see (B) in Supplementary Table 3), where it would obtain a slightly lower centrality (0.224) but could benefit from other potential advantages of being in connection to strong research groups.

Note that this is just a toy model and real cases should be evaluated taking into account more precise co-authorship networks, as well as other socio-economical aspects. Nevertheless, we believe that this methodology could be an additional tool to help a research centre making a correct decision. Finally, let us remark that similar studies could guide national policy makers when promoting collaborations between scientists of different countries, where, in this case, each competing network accounts for collaborations among researchers of a particular country and the creation of links is supported by grants promoting collaboration among countries (e.g. the bilateral programs for improving scientific collaboration among members of the European Union).

Technological networks

◦ Multi-layer air transportation networks

Although social networks are possibly the ones that can benefit the most from the methodology proposed in this paper, applications to technological networks can also be of interest. For example, air transportation networks rely on the coordination of different flying companies, which have the possibility of creating connections among them in order to attract a higher number of tentative customers. Recently, air transportation networks have been interpreted as multi-layer networks, where layers account for different commercial airlines, showing that the projection into a multi-layer structure describes more precisely the topological properties of air transportation networks [16]. From this point of view, we can create a network-of-networks where airlines are subnetworks interacting through an exchange of passengers at certain airports, being the latter the fundamental nodes of the air transportation network and a flight between two airports giving rise to a link between two nodes. As in [16], this point of view leads to a multi-layer network, where each layer accounts for the connections inside a given airline. Next, we can create the inter-layer connections when two companies decide to sign an agreement, exchanging passengers at certain airports (see Supplementary Fig. 4 for a qualitative description). Now the problem is how an airline could choose the most adequate airline to connect to, taking into account that other companies are competitors that also try to maximize their own benefit. Using the eigenvector centrality as a

measure of importance for the airports [17], our methodology could reveal the most adequate partners to deal with.

Importantly, the rules and equilibria predicted by the methodology proposed in this paper fully apply to this particular case and to multi-layer networks in general.

Biological networks

◦ Connecting fragmented habitats

The application of the proposed methodology to biology is more limited than in the case of social or technological networks, since most biological networks, such as protein interaction or gene regulatory networks, lack the ability to choose among different strategies of connection. However, here we present one special case to motivate the reader to search for other applications in this field.

We focus on the problem of fragmented habitats, in which certain species live in close but sparsely connected locations (also known as patches). The connectivity of a landscape is a critical factor for the persistence of flora and fauna, the maintenance of high levels of genetic variability, and the adaptability of species to environmental changes [18]. Within this framework, network theory is often applied to evaluate the degree of fragmentation of a habitat and to design efficient conservation strategies [19]. In a network representation of a landscape the nodes correspond to patches of conserved habitat where a species can survive, and the links are routes (e.g. ecological corridors, wildlife crossings. . .) through which individuals can disperse from one patch to another.

Entities engaged in species conservation and landscape management can mitigate the negative effects of habitat fragmentation by creating new connections among patches. However, it is not unusual that the distribution range of a species spans over lands administered by different entities, which calls for coordinated actions for the conservation of the species. An example of this situation could be the intensive work developed during the last decades to reintroduce and protect the brown bear (*Ursus arctos*) in the Pyrenees, a challenge addressed in coordination among the governments of France, Spain and Andorra. Other example would be the acquisition of forest lands in endangered areas of the African equatorial forest or the Amazonia by different non-profit organizations with the aim of fighting deforestation. Because the creation and maintenance of ecological corridors requires a significant investment, how should areas managed by different countries/organizations be connected, if each participant aspires to maximize the outcome (e.g. biodiversity) in the area it manages?

This could be a good example to use the alternative payoff explained at the beginning of Supplementary Note 5, where competitors try to maximize the eigenvector centrality multiplied by the maximum eigenvalue. In this case, the eigenvector would be proportional to the population of the species and its associated eigenvalue would be a proxy for the growth rate of the species, a quantity that in this case should also be considered. As we show in that Supplementary Note, the phenomenology is the same as in the general case studied in the manuscript, and therefore depending on the size and ecological potential of each portion of the habitat (i.e. network) the stable ways to connect them would be: (i) the connection of weak networks with the strong one (X_∞), or (ii) the collaboration between the weak (X_0). Note that, as mentioned in the manuscript and shown at the end of Supplementary Note 3, the final global eigenvalue of solution X_0 is always larger than that of X_∞, which means that the final growth rate of the endangered species after the connection would be optimized if the different networks decided to collaborate.

Supplementary Note 2. Basic aspects of the competition among networks for eigenvector centrality

The election of eigenvector centrality as the metric to be optimized

The quantification of the importance of a node within a network is an issue with a long tradition, and the eigenvector centrality is the most extended metric due to its practical applications. To name a few, it is behind the Google Pagerank algorithm, which is an indicator of the importance of a given webpage and, in turn, it is used to elaborate the lists of webpages displayed by Google when doing a certain search [20]. The idea of using the eigenvector centrality to evaluate the scientific impact of scientists has also been proposed [21] and the eigenfactor project [22] has recently adapted the eigenvector centrality to be an effective measure for quantifying Journal importance. Other illustrative (real) applications of the eigenvector centrality are the detection of important regions in the brain [23] or the, more classical, evaluation of importance of an individual within a social group [24]. In addition, the eigenvector centrality is also related with a diversity of dynamical processes such as disease or rumour spreading (see [2] for an overview).

Relevant definitions and analytical solution of the two-network problem

The competition for eigenvector centrality between two networks A and B that are connected through a small number of *connector links* was established and analysed in [25]. We review here the main conclusions.

Networks A and B have N_A and N_B nodes respectively. We connect them through L connector links to create a new network T of $N_T = N_A + N_B$ nodes. For simplicity, let us suppose that A and B are weighted but undirected networks.

Let us call $\lambda_{A,i}$, and $\lambda_{B,i}$ the i eigenvalues of the adjacency matrices $\mathbf{G_A}$ and $\mathbf{G_B}$ respectively. Let us suppose $\lambda_{A,1} > \lambda_{B,1}$. We call $\vec{u}_{A,i}$ to the N_A eigenvectors of network A, and $\vec{u}_{B,i}$ to the N_B eigenvectors of network B.

The eigenvector $\vec{u}_{T,1}$ determines the outcome of the competition between networks A and B. Centrality A (C_A) and centrality B (C_B) are the fractions of the total centrality that remain in the nodes of network A and B after the connection, and are obtained as

$$C_A = \frac{\sum_{i=1}^{N_A} (\vec{u}_{T,1})_i}{\sum_{i=1}^{N_T} (\vec{u}_{T,1})_i}, \quad (1)$$

$$C_B = 1 - C_A . \quad (2)$$

This way, the goal of the competition between networks is to increase C as much as possible. The first eigenvalue and eigenvector of the total network T formed by the networks A and B connected, expressed as quantities that are only dependent on the isolated networks A and B, can be approximated to first order by

$$\vec{u}_{T,1} = \vec{u}_{A,1} + \epsilon \sum_{k=1}^{N_B} \frac{\vec{u}_{A,1}\mathbf{P}\vec{u}_{B,k}}{\lambda_{A,1}-\lambda_{B,k}} \vec{u}_{B,k} + o(\epsilon^2) , \quad (3)$$

$$\lambda_{T,1} = \lambda_{A,1} + \epsilon^2 \sum_{k=1}^{N_B} \frac{(\vec{u}_{A,1}\mathbf{P}\vec{u}_{B,k})^2}{\lambda_{A,1}-\lambda_{B,k}} + o(\epsilon^3) . \quad (4)$$

Note that $P_{ij} = P_{ji} = 1$ for $ij \in \{cl\}$ and $P_{ij} = 0$ elsewhere, being $\{cl\}_{i=1,\ldots,L}$ the set of pairs lm corresponding to the connector links that attach the connector nodes l of network A with nodes m of network B to form T. Furthermore, the terms $k = 1$ of both summations are the most relevant ones because $\lambda_{A,1} - \lambda_{B,k} > \lambda_{A,1} - \lambda_{B,1}$ for $k > 1$. Furthermore, $\vec{u}_{A,1}\mathbf{P}\vec{u}_{B,1} = \sum_{cl} (\vec{u}_{A,1})_i (\vec{u}_{B,1})_j$, that is, the sum of the products of the eigenvector centralities of all connector nodes measured when the networks are disconnected.

For these reasons, we obtain

$$\vec{u}_{T,1} \sim \frac{\vec{u}_{A,1}+\epsilon\frac{\sum_{cl}(\vec{u}_{A,1})_i(\vec{u}_{B,1})_j}{\lambda_{A,1}-\lambda_{B,1}}\vec{u}_{B,1}}{\sqrt{1+\epsilon^2\left(\frac{\sum_{cl}(\vec{u}_{A,1})_i(\vec{u}_{B,1})_j}{\lambda_{A,1}-\lambda_{B,1}}\right)^2}}\,, \quad (5)$$

$$\lambda_{T,1} \sim \lambda_{A,1}+\epsilon^2\frac{[\sum_{cl}(\vec{u}_{A,1})_i(\vec{u}_{B,1})_j]^2}{\lambda_{A,1}-\lambda_{B,1}}\,, \quad (6)$$

where $\vec{u}_{T,1}$ has been normalized such that $|\vec{u}_{T,1}| = 1$. Note that $\vec{u}_{T,1}$, $\vec{u}_{A,1}$ and $\vec{u}_{B,1}$ are vectors of length $N_A + N_B$ and norm $|\vec{u}_{A,1}| = |\vec{u}_{B,1}| = |\vec{u}_{T,1}| = 1$. The first N_A elements of $\vec{u}_{A,1}$ are equal to the first eigenvector of network A isolated and its last N_B elements are equal to zero, while the first N_A elements of $\vec{u}_{B,1}$ are equal to zero and its last N_B elements are equal to the first eigenvector of network B isolated.

In conclusion, the analysis of Eqs. (5-6) yields that the distribution of the centrality in a competition between two networks is critically dependent on the *connector nodes*, that is, those connected by the *connector links*, as well as on the largest eigenvalues $\lambda_{A,1}$ and $\lambda_{B,1}$ of the isolated networks A and B.

Successful strategies

In practice, unless $\lambda_{A,1}$ and $\lambda_{B,1}$ are almost equal, the network with the highest eigenvalue always obtains a higher centrality. This enables us to call *strong* network to the one with the largest first eigenvalue, and *weak* network to the other one.

We call *central node* of a network to the node that corresponds to the largest element of the eigenvector associated with the first eigenvalue of the adjacency matrix of such network. The *peripheral nodes* are those whose centrality is very low in relation to that of the central one.

Following Eqs. (3-4) or Eqs. (5-6), the optimal strategies for the players become:

1. Connecting the most central nodes of two networks optimizes centrality of the weak network.

2. Connecting the most peripheral nodes of two networks optimizes centrality of the strong network.

3. Increasing the number of connector links reinforces centrality of the weak network.

4. Any variation that increases the maximum eigenvalue associated to a network (e.g. suitable internal rewiring, addition of nodes or links, etc.) increases its centrality.

Selection of the specific connector nodes

Regarding the former subsection, it is clear that the initial centrality of the connector nodes (i.e., the centrality before connecting networks between them) is crucial in the final distribution of centrality in the network-of-networks. When connecting two networks, the connector nodes allow to distinguish between central-central (CC) connections or peripheral-peripheral (PP) connections. PP connections lead to a scenario close to the disconnected case, thus pushing almost the totality of the centrality towards the strong network (see *Methods* in the main manuscript). This fact is illustrated, by means of an example, in Supplementary Fig. 5. We have selected the Nash equilibria obtained in the example of the main text (i.e., that of the three Indian villages) and modified the connector links to join the peripheral nodes of the three networks (i.e., to have PP connections between them), instead of the central nodes. In both solutions X_0 and X_∞, when links connect peripheral nodes, the distribution of centrality is very close to the isolated case (i.e, in the absence of links between networks), leading the network with the highest λ_1 to retain the centrality of the whole system (see table of Supplementary Fig. 5 for the actual values of centrality).

For these reasons, in the rules of the competition we have restricted the connections to (i) the CC case, that is, the connector links between networks always connect the central nodes of such networks, and to (ii) the absence of connection, which in practice coincides with a PP connection. This fact should be taken into account in both the numerical and the analytical work shown in this paper.

Supplementary Note 3. Analytical study of the competition among three networks

Alternative definition for the eigenvector centrality in competition among m networks

In the main manuscript and the numerical work we have measured the eigenvector centrality of each network i as C_i (see Eqs. (1-2)). For simplicity, in the analytical treatment of the phenomenology we will use an alternative definition of the centrality when m networks compete: $\mathcal{C}_i$. It is based on Eqs. (3) or (5), from where we obtain that the eigenvector of the connected system can be approximated as

$$\vec{u}_{T,1} \sim \sum_{i=1}^{m} \mathcal{C}_i \vec{u}_{i,1} \tag{7}$$

where $\sum_{i=1}^{m} \mathcal{C}_i^2 = 1$, $0 \le \mathcal{C}_i \le 1$ for all i, $|\vec{u}_{i,1}| = 1$ for all i, and $|\vec{u}_{T,1}| = 1$.

Let us see that both definitions C_i and $\mathcal{C}_i$ are qualitatively equivalent. The relation between C_i and $\mathcal{C}_i$ is

$$C_i = \frac{\sum_{j_i} (\vec{u}_{T,1})_{j_i}}{\sum_{k=1}^{N_T} (\vec{u}_{T,1})_k} = \frac{\mathcal{C}_i \sum_{j_i} (\vec{u}_{i,1})_{j_i}}{\sum_{k=1}^{m} \mathcal{C}_k \sum_{j_k} (\vec{u}_{k,1})_{j_k}} \,,$$

where j_i are the nodes that belong to network i (the same stands for j_k). Note that (i) $\sum_{j_i} (\vec{u}_{i,1})_{j_i} > 0$ for all i, $\sum_{j_k} (\vec{u}_{k,1})_{j_k} > 0$ for all k, and both terms are constant during the competition because they do not depend on how the networks are inter-connected. From here we obtain that $C_i = 0 \iff \mathcal{C}_i = 0$; (ii) $C_i = 1 \Rightarrow \mathcal{C}_k \sum_{j_k} (\vec{u}_{k,1})_{j_k} = 0$ for all $k \neq i$ which yields that $C_i = 1 \iff \mathcal{C}_i = 1$; and (iii)

$$\frac{\partial C_i}{\partial \mathcal{C}_i} = \frac{\sum_{j_i} (\vec{u}_{i,1})_{j_i}}{\sum_{k=1}^{m} \mathcal{C}_k \sum_{j_k} (\vec{u}_{k,1})_{j_k}} \left(1 - \frac{\mathcal{C}_i \sum_{j_i} (\vec{u}_{i,1})_{j_i}}{\sum_{k=1}^{m} \mathcal{C}_k \sum_{j_k} (\vec{u}_{k,1})_{j_k}} \right) = \frac{C_i}{\mathcal{C}_i} (1 - C_i) > 0$$

for $C_i < 1$ from where we obtain that $\mathcal{C}_i$ grows monotonically with C_i, for all i. From (i-iii) we obtain that C_i and $\mathcal{C}_i$ are equivalent to describe eigenvector centrality. However, the use of $\mathcal{C}_i$ will be of special use to develop analytical work, because it avoids using quantities $\sum_j (\vec{u}_{i,1})_j$ (that will depend on the peculiarities of each network $i = A, B, ...$) and benefits from the fact that $|\vec{u}_{i,1}| = 1$ for all i.

Basic methodology to analytically prove that a configuration is a Nash equilibrium

For the sake of precision and simplicity, in this work we have detected numerically the Nash equilibria in the different configurations that we have studied. However, in a wide variety of cases it is possible to analytically prove whether or not a configuration is a Nash equilibrium. Here we present a methodology to do it.

In the competition among three networks, and when each competitor can create as much as one connector link ($l = 1$) between the central nodes of the different networks, competitor X can follow three different connection strategies in relation to its competitors Y and Z:

- $X(0)$: Network X decides not to connect to any other network.
- $X \to Y$: Network X connects to network Y. Note that the symbol $\to$ is used to remark which network decides to create that link, but the connector link is undirected.
- $X \to Z$: Network X connects to network Z.

This gives rise to $3 \times 3 \times 3 = 27$ different configurations, only a few of them being Nash equilibria. To verify by analytical means whether a configuration of three networks is a Nash equilibrium, one should check how the centrality of the competitors change when they vary their connection strategy. Only when all changes in the connection strategies of each competitor lead to loss in centrality, will we face a Nash equilibrium. In order to calculate such variation of centrality, we present here a methodology based on regarding two of the three networks as a single one that competes against the remaining network, and apply the following *conditions* 1, 2 or 3 depending on how they are connected:

1. $X\ Y$: Network X isolated form network Y: $\lambda_{X,1} > \lambda_{Y,1} \Rightarrow \mathcal{C}_X = 1, \mathcal{C}_Y = 0$.

2. $X \leftrightarrow Y$: Network X connected with network Y through 1 link. If $\lambda_{X,1} > \lambda_{Y,1}$:

$$\mathcal{C}_X \sim \frac{1}{\sqrt{1+\epsilon^2\left[\frac{\sum_{cl}(\vec{u}_{X,1})_i(\vec{u}_{Y,1})_j}{\lambda_{X,1}-\lambda_{Y,1}}\right]^2}},$$

$$\mathcal{C}_Y = \sqrt{1-\mathcal{C}_X^2} \sim \frac{\epsilon\frac{\sum_{cl}(\vec{u}_{X,1})_i(\vec{u}_{Y,1})_j}{\lambda_{X,1}-\lambda_{Y,1}}}{\sqrt{1+\epsilon^2\left[\frac{\sum_{cl}(\vec{u}_{X,1})_i(\vec{u}_{Y,1})_j}{\lambda_{X,1}-\lambda_{Y,1}}\right]^2}}.$$

3. $X \leftrightarrows Y$: Network X connected with network Y through 2 links. If $\lambda_{X,1} > \lambda_{Y,1}$:

$$\mathcal{C}_X \sim \frac{1}{\sqrt{1+4\epsilon^2\left[\frac{\sum_{cl}(\vec{u}_{X,1})_i(\vec{u}_{Y,1})_j}{\lambda_{X,1}-\lambda_{Y,1}}\right]^2}},$$

$$\mathcal{C}_Y = \sqrt{1-\mathcal{C}_X^2} \sim \frac{2\epsilon\frac{\sum_{cl}(\vec{u}_{X,1})_i(\vec{u}_{Y,1})_j}{\lambda_{X,1}-\lambda_{Y,1}}}{\sqrt{1+4\epsilon^2\left[\frac{\sum_{cl}(\vec{u}_{X,1})_i(\vec{u}_{Y,1})_j}{\lambda_{X,1}-\lambda_{Y,1}}\right]^2}}.$$

From here we obtain that, for $\lambda_{X,1} > \lambda_{Y,1}$, $1 = \mathcal{C}_X(X\ Y) > \mathcal{C}_X(X \leftrightarrow Y) > \mathcal{C}_X(X \leftrightarrows Y)$, and $0 = \mathcal{C}_Y(X\ Y) < \mathcal{C}_Y(X \leftrightarrow Y) < \mathcal{C}_X(X \leftrightarrows Y)$, as it could be obtained from the strategies presented in the subsection *Successful strategies* of Supplementary Note 3.

Analytical study of a remarkable example: Configuration X_∞

For clarity, let us show the methodology just proposed to show that configuration $X_\infty = \{A(0), B \rightarrow A, C \rightarrow A\}$ is a Nash equilibrium.

- Network A: Its current strategy is $A(0)$.
  - $A \rightarrow B$ would lead to $X'_\infty = \{A \leftrightarrows B, C \rightarrow A\}$, that is, strong network A doubles its connection to weak network B (i.e. it changes condition 2 by condition 3 presented in the former subsection) and therefore $\mathcal{C}_\mathcal{A}(X_\infty) < \mathcal{C}_\mathcal{A}(X'_\infty)$.
  - $A \rightarrow C$ would lead to $X'_\infty = \{A \leftrightarrows C, B \rightarrow A\}$, and happens as in the former case.
- Network B: Its current strategy is $B \rightarrow A$.
  - $B(0)$ would lead to $X'_\infty = \{A(0), B(0), C \rightarrow A\}$, that is, weak network B isolates from the rest and leads to $\mathcal{C}_\mathcal{B}(X'_\infty) = 0 < \mathcal{C}_\mathcal{B}(X_\infty)$.

  - $B \rightarrow C$ would lead to $X'_\infty = \{A(0), B \rightarrow C, C \rightarrow A\}$, that is, weak network B stops connecting to the most central node of the strong network formed by $A \leftrightarrow C$ (which is the most beneficial strategy) and connects to a weaker central node in C. Therefore $\mathcal{C}_\mathcal{B}(X'_\infty) = 0 < \mathcal{C}_\mathcal{B}(X_\infty)$.

- Network C: Its current strategy is $C \rightarrow A$.
  - $C(0)$ would lead to $X'_\infty = \{A(0), B \rightarrow A, C(0)\}$, that is, weak network C isolates from the rest and leads to $\mathcal{C}_\mathcal{C}(X'_\infty) = 0 < \mathcal{C}_\mathcal{C}(X_\infty)$.
  - $C \rightarrow B$ would lead to $X'_\infty = \{A(0), B \rightarrow A, C \rightarrow B\}$, that is, weak network C stops connecting to the most central node of the strong network formed by $A \leftrightarrow B$ (which is the most beneficial strategy) and connects to a weaker central node in B. Therefore $\mathcal{C}_\mathcal{C}(X'_\infty) = 0 < \mathcal{C}_\mathcal{C}(X_\infty)$.

In conclusion, all existing possibilities of changing the strategy lead to worse final payoffs for the competitor who developed those changes, and therefore configuration $X_\infty = \{A(0), B \rightarrow A, C \rightarrow A\}$ is a Nash equilibrium. The same methodology could be used to prove that other configurations do or do not verify the conditions to be Nash equilibria.

Proof of the existence of a critical transition at $\lambda_{A,1} = \lambda_{B\leftrightarrows C,1}$

As can be seen in Fig. 2 of the main manuscript, for $\lambda_{A,1} > \lambda_{B\leftrightarrows C,1}$ the only remaining Nash equilibrium is $X_\infty = \{A(0), B \rightarrow A, C \rightarrow A\}$. Using the methodology presented above to analyse the phenomenology here studied, it is easy to prove that configuration $X_0 = \{A \rightarrow B, B \leftrightarrows C\}$ stops being a Nash equilibrium critically at $\lambda_{A,1} = \lambda_{B\leftrightarrows C,1}$.

- Network A: Its strategy in X_0 is $A \rightarrow B$.
  - $A(0)$ would lead to $X'_0 = \{A(0), B \leftrightarrows C\}$. Let us face the system as a competition between network A and network $B \leftrightarrows C$. In regime $\lambda_{A,1} < \lambda_{B\leftrightarrows C,1}$, network A isolates from the strong network $B \leftrightarrows C$, and following condition 1 presented in the subsection *Basic methodology to analytically prove that a configuration is a Nash equilibrium*, results in $\mathcal{C}_\mathcal{A}(X'_0) = 0 < \mathcal{C}_\mathcal{A}(X_0)$, which is detrimental for A, a fact that is compatible with a Nash equilibrium. On the other hand, for $\lambda_{A,1} \geq \lambda_{B\leftrightarrows C,1}$ network A becomes a stronger network than $B \leftrightarrows C$, and therefore condition 1 yields $\mathcal{C}_\mathcal{A}(X'_0) = 1 > \mathcal{C}_\mathcal{A}(X_0)$, independently of the value of $\mathcal{C}_\mathcal{A}(X_0)$. In conclusion, for $\lambda_{A,1} > \lambda_{B\leftrightarrows C,1}$ network A becomes so strong that will always tend to isolate from B and C, and therefore the configuration X_0 is not a Nash equilibrium in that regime.

On the other hand, configuration X_∞ is not affected by this transition, as the proof shown in the former subsection is also valid for large values of $\lambda_{A,1}$.

Proof of $C_A(X_\infty) > C_A(X_0)$

Here we prove that, for networks of all size and topologies, configuration $X_\infty = \{A(0), B \rightarrow A, C \rightarrow A\}$ is more beneficial for the strong network A than configuration $X_0 = \{A \rightarrow B, B \leftrightarrows C\}$. The contrary stands for the weak networks B and C. Note that X_0 can be a Nash equilibrium only in $\lambda_{A,1} < \lambda_{B\leftrightarrows C,1}$, and therefore we restrict our proof to that regime.

If we extend Eq. 5 for three networks following configuration X_∞, we have

$$\vec{u}_{T,1}(X_\infty) \sim \frac{\vec{u}_{A,1} + \epsilon \frac{\sum_{cl} (\vec{u}_{A,1})_i (\vec{u}_{B,1})_j}{\lambda_{A,1} - \lambda_{B,1}} \vec{u}_{B,1} + \epsilon \frac{\sum_{cl} (\vec{u}_{A,1})_i (\vec{u}_{C,1})_j}{\lambda_{A,1} - \lambda_{C,1}} \vec{u}_{C,1}}{\sqrt{1 + \epsilon^2 \left(\frac{\sum_{cl} (\vec{u}_{A,1})_i (\vec{u}_{B,1})_j}{\lambda_{A,1} - \lambda_{B,1}} \right)^2 + \epsilon^2 \left(\frac{\sum_{cl} (\vec{u}_{A,1})_i (\vec{u}_{C,1})_j}{\lambda_{A,1} - \lambda_{C,1}} \right)^2}}$$

while in configuration X_0, we have

$$\vec{u}_{T,1}(X_0) \sim \frac{\vec{u}_{B\leftrightarrows C,1} + \epsilon \frac{\sum_{cl} (\vec{u}_{B\leftrightarrows C,1})_i (\vec{u}_{A,1})_j}{\lambda_{B\leftrightarrows C,1} - \lambda_{A,1}} \vec{u}_{A,1}}{\sqrt{1 + \epsilon^2 \Big(\frac{\sum_{cl} (\vec{u}_{B\leftrightarrows C,1})_i (\vec{u}_{A,1})_j}{\lambda_{B\leftrightarrows C,1} - \lambda_{A,1}} \Big)^2}}$$

where

$$\vec{u}_{B\leftrightarrows C,1}(X_0) \sim \frac{\vec{u}_{B,1} + \epsilon \frac{\sum_{cl} (\vec{u}_{B,1})_i (\vec{u}_{C,1})_j}{\lambda_{B,1} - \lambda_{C,1}} \vec{u}_{C,1}}{\sqrt{1 + \epsilon^2 \Big(\frac{\sum_{cl} (\vec{u}_{B,1})_i (\vec{u}_{C,1})_j}{\lambda_{B,1} - \lambda_{C,1}} \Big)^2}} \,,$$

$$\lambda_{B\leftrightarrows C,1} = \lambda_{B,1} + 4\epsilon^2 \frac{[\sum_{cl} (\vec{u}_{B,1})_i (\vec{u}_{C,1})_j]^2}{\lambda_{B,1} - \lambda_{C,1}} \,.$$

Our aim is to prove that $\mathcal{C}_{\mathcal{A}}(X_\infty) > \mathcal{C}_{\mathcal{A}}(X_0)$, that is,

$$\frac{1}{\sqrt{1 + \epsilon^2 \Big(\frac{\sum_{cl} (\vec{u}_{A,1})_i (\vec{u}_{B,1})_j}{\lambda_{A,1} - \lambda_{B,1}} \Big)^2 + \epsilon^2 \Big(\frac{\sum_{cl} (\vec{u}_{A,1})_i (\vec{u}_{C,1})_j}{\lambda_{A,1} - \lambda_{C,1}} \Big)^2}} > \frac{\epsilon \frac{\sum_{cl} (\vec{u}_{B\leftrightarrows C,1})_i (\vec{u}_{A,1})_j}{\lambda_{B\leftrightarrows C,1} - \lambda_{A,1}}}{\sqrt{1 + \epsilon^2 \Big(\frac{\sum_{cl} (\vec{u}_{B\leftrightarrows C,1})_i (\vec{u}_{A,1})_j}{\lambda_{B\leftrightarrows C,1} - \lambda_{A,1}} \Big)^2}}$$

from where we obtain that

$$\epsilon^4 \left(\frac{\sum_{cl} (\vec{u}_{B\leftrightarrows C,1})_i (\vec{u}_{A,1})_j}{\lambda_{B\leftrightarrows C,1} - \lambda_{A,1})} \right)^2 \left[\left(\frac{\sum_{cl} (\vec{u}_{A,1})_i (\vec{u}_{B,1})_j}{\lambda_{A,1} - \lambda_{B,1}} \right)^2 + \left(\frac{\sum_{cl} (\vec{u}_{A,1})_i (\vec{u}_{C,1})_j}{\lambda_{A,1} - \lambda_{C,1}} \right)^2 \right] < 1 \,. \tag{8}$$

Taking into account that

$$\sum_{cl} (\vec{u}_{B\leftrightarrows C,1})_i (\vec{u}_{A,1})_j = \vec{u}_{B\leftrightarrows C,1} \mathbf{P} \vec{u}_{A,1} = \frac{\vec{u}_{B,1} \mathbf{P} \vec{u}_{A,1} + 2\epsilon \frac{(\vec{u}_{B,1} \mathbf{P} \vec{u}_{C,1})(\vec{u}_{C,1} \mathbf{P} \vec{u}_{A,1})}{\lambda_{B,1} - \lambda_{C,1}}}{\sqrt{1 + 4\epsilon^2 (\frac{\vec{u}_{B,1} \mathbf{P} \vec{u}_{C,1}}{\lambda_{B,1} - \lambda_{C,1}})^2}}$$

and $\vec{u}_{C,1} \mathbf{P} \vec{u}_{A,1} = 0$ because networks A and C are not connected in configuration X_0, we obtain

$$\sum_{cl} (\vec{u}_{B\leftrightarrows C,1})_i (\vec{u}_{A,1})_j = \frac{\sum_{cl} (\vec{u}_{A,1})_i (\vec{u}_{B,1})_j + 2\epsilon \frac{\sum_{cl} (\vec{u}_{B,1})_i (\vec{u}_{C,1})_j \sum_{cl} (\vec{u}_{C,1})_i (\vec{u}_{A,1})_j}{\lambda_{B,1} - \lambda_{C,1}}}{\sqrt{1 + 4\epsilon^2 \Big[\frac{\sum_{cl} (\vec{u}_{B,1})_i (\vec{u}_{C,1})_j}{\lambda_{B,1} - \lambda_{C,1}} \Big]^2}} \,. \tag{9}$$

Introducing Eq. 9 in Eq. 8 and developing in powers of ϵ, we finally obtain that the condition that should be fulfilled to verify $\mathcal{C}_{\mathcal{A}}(X_\infty) > \mathcal{C}_{\mathcal{A}}(X_0)$ is

$$\xi = \epsilon^4 \left(\frac{\sum_{cl} (\vec{u}_{A,1})_i (\vec{u}_{B,1})_j}{\lambda_{A,1} - \lambda_{B,1}} \right)^2 \left[\left(\frac{\sum_{cl} (\vec{u}_{A,1})_i (\vec{u}_{B,1})_j}{\lambda_{A,1} - \lambda_{B,1}} \right)^2 + \left(\frac{\sum_{cl} (\vec{u}_{A,1})_i (\vec{u}_{C,1})_j}{\lambda_{A,1} - \lambda_{C,1}} \right)^2 \right] < 1 \,.$$

According to Eq. (5), the terms $\frac{\sum_{cl} (\vec{u}_{A,1})_i (\vec{u}_{B,1})_j}{\lambda_{A,1} - \lambda_{B,1}}$ and $\frac{\sum_{cl} (\vec{u}_{A,1})_i (\vec{u}_{C,1})_j}{\lambda_{A,1} - \lambda_{C,1}}$ represent the fraction of centrality in the weak networks B and C in the two-network systems formed by A connected to B and A connected to C respectively. As A is the strongest network (that is, as $\lambda_{A,1} > \lambda_{B,1} > \lambda_{C,1}$), both expressions are in general much smaller than 1. Therefore, for small values of the weight of the connector links ϵ, it is clear that $\xi < 1$ and $\mathcal{C}_{\mathcal{A}}(X_\infty) > \mathcal{C}_{\mathcal{A}}(X_0)$.

Supplementary Note 4. Analysis of the total strength of systems composed of three competing networks: an illustrative example

Throughout this work we have focused on the competition among interconnected networks of different strength, being such strength measured as the maximum eigenvalue λ_1 of the adjacency matrix associated to each network. But what happens if we study the strength of the whole system formed by the network-of-networks?

Extensive numerical work summarized in Supplementary Fig. 6 yields that the strength associated to a system made of 3 interconnected networks following the *cooperative* equilibrium $X_0 = \{A \to B, B \leftrightarrows C\}$ is always larger than that of the *alliance with the strong* solution $X_\infty = \{A(0), B \to A, C \to A\}$ (i.e., $\lambda_1(X_0) > \lambda_1(X_\infty)$) in the range of parameters in which both are Nash equilibria ($\lambda_{A,1} < \lambda_{B\leftrightarrows C,1}$). This fact can have important consequences, as λ_1 represents the growth at the equilibrium for a wide range of dynamical processes [26], among some other quantities related to synchronizability or percolation. In summary, λ_1 can be treated as a proxy for efficiency and growth.

Taking all this into account, the phenomenology presented here could yield a new perspective to those sociological, economical or technological situations where a system based on the submission to a powerful leader led to a more stable and efficient organization based on cooperation. With the only aim of illustrating the methodology, and being aware of the complexity associated to the example here analysed, let us show that our novel point of view is able to reproduce the main highlights of a paradigmatic historical transition: the gradual dissolution of the European medieval system based on the feudal vassalage when, during the 12th-14th centuries, the guilds and professional associations gained importance and created a new attracting pole in contrast to the countryside controlled by feudal lords, the city [29, 30]. As a consequence of the promotion of the urban environment, social and professional connections were largely strengthened, being the highly connected association between the weak networks shown in steps 1 and 2 in Fig.3a of the main text a very simplified scheme of this phenomenon. The concomitant increase in the flow of goods and currency gave rise to a parallel enhancement of the standard of economy and living in towns (represented in our model by the raise of the strength of the whole network-of-networks, as we know from Supplementary Fig. 6 that $\lambda_1(X_0) > \lambda_1(X_\infty)$). This fact eventually forced the nobility, now in a clear disadvantageous situation due to the loss of importance of the rural areas, to definitely incline towards cities and royal courts (step 3 in Fig.3a of the main text), contributing to the twilight of the feudal system and the consequent consolidation of cities in Europe that gave way to Renaissance.

Supplementary Note 5. Robustness of the results

The phenomenology can be generalized to other configurations of competing networks. Besides the modifications presented in the main manuscript, here we analyse briefly the cases in which different definitions of the payoffs are used, the topology of the networks is not scale-free, the competitors can make use of different number/weight of connector nodes, mixed connection strategies are allowed, the size of the networks is very small, and finally the case in which there are more than three networks in the competition.

Competition with different definitions of the payoffs

Alternative definitions for the payoff in the competition could be of interest depending on the nature of the system under study. Here we study (i) a payoff equal to the eigenvector centrality multiplied by the total eigenvalue $\lambda_{T,1}$ of the system to take into account the improvement of the growth of each node, (ii) the competition for betweenness centrality, and (iii) the competition for closeness centrality.

◦ Competition for the eigenvector centrality multiplied by the total eigenvalue of the system

An especially interesting example,

$$\mathcal{C}'_i = \lambda_{T,1}\mathcal{C}_i\,, \tag{10}$$

takes into account both the distribution in the equilibrium of a certain population that evolves over the network, represented by $\vec{u}_{T,1}$, and the growth of such population at the equilibrium given by $\lambda_{T,1}$. Therefore, multiplying the centrality by $\lambda_{T,1}$ includes in the definition of the payoff the improvement of the growth of each node. This can be useful when, for example, economical dynamics among companies are being modelled: when two or more companies merge, it is plausible to think that the whole new business accelerates its growth rate, and this positive effect should be weighted in the payoff. Another interesting example could be that of the connection of fragmented habitats, where the growth rate of the different species is a critical quantity to optimize (see the last subsection of Supplementary Note 1 for more details on the modelization of fragmented habitats).

It is known that $\mathcal{C}_\mathcal{A}$ decreases with the weight of the connector links ϵ or the value of the eigenvector at the connector nodes $\sum_{cl} (\vec{u}_{A,1})_l (\vec{u}_{B,1})_m$, while $\lambda_{T,1}$ grows with them (see Eqs. (3-4) and the subsection *Successful strategies* of Supplementary Note 3). While this fact could make us think that the response of this new way to measure the contest could be different to that already studied for C_i and $\mathcal{C}_i$, we prove here that the phenomenology of competition and the relating strategies shown in Supplementary Note 3 are not varied with this alternative definition of the eigenvector centrality.

Let us suppose two networks A and B that compete for centrality defined by Eq. 7,

$$\vec{u}_{T,1} \sim \mathcal{C}_\mathcal{A}\vec{u}_{A,1} + \mathcal{C}_\mathcal{B}\vec{u}_{B,1}\,.$$

Including Eqs. (5) and (6) in Eq. (10), and developing in series of ϵ, we obtain

$$\mathcal{C}_{\mathcal{A}} \sim \frac{1}{\sqrt{1+\left(\epsilon\frac{\sum_{cl}(\vec{u}_{A,1})_i(\vec{u}_{B,1})_j}{\lambda_{A,1}-\lambda_{B,1}}\right)^2}} \sim 1-\frac{1}{2}\left(\frac{\sum_{cl}(\vec{u}_{A,1})_i(\vec{u}_{B,1})_j}{(\lambda_{A,1}-\lambda_{B,1})}\right)^2\epsilon^2+o(\epsilon^3)\,, \tag{11}$$

$$\begin{aligned}\mathcal{C}'_{\mathcal{A}} &= \lambda_{T,1}\mathcal{C}_{\mathcal{A}} \sim \frac{\lambda_{A,1}+\epsilon^2\frac{[\sum_{cl}(\vec{u}_{A,1})_i(\vec{u}_{B,1})_j]^2}{\lambda_{A,1}-\lambda_{B,1}}}{\sqrt{1+\left(\epsilon\frac{\sum_{cl}(\vec{u}_{A,1})_i(\vec{u}_{B,1})_j}{\lambda_{A,1}-\lambda_{B,1}}\right)^2}}\\ &\sim \lambda_{A,1}-\frac{(2\lambda_{B,1}-\lambda_{A,1})[\sum_{cl}(\vec{u}_{A,1})_i(\vec{u}_{B,1})_j]^2}{2(\lambda_{A,1}-\lambda_{B,1})^2}\epsilon^2+o(\epsilon^3)\,. \end{aligned}\tag{12}$$

As it was already known, the traditional definitions of centrality C_A or $\mathcal{C}_{\mathcal{A}}$ decrease when either the weight of the connector links ϵ or the value of the eigenvector at the connector nodes $\sum_{cl}(\vec{u}_{A,1})_i(\vec{u}_{B,1})_j$ increase (Eq.(11)). As we can see in Eq. (12), $\mathcal{C}'_{\mathcal{A}} = \lambda_{T,1}\mathcal{C}_{\mathcal{A}}$ also decreases with such quantities for $\lambda_{A,1} < 2\lambda_{B,1}$, which represents all relevant cases. Note that $\lambda_{A,1} > 2\lambda_{B,1}$ means a trivial competition in which the difference between both networks is so large that virtually all of the centrality remains in network A, regardless of the strategy followed by the weak network.

In summary, the phenomenology of a competition between two networks for centrality defined as $\mathcal{C}'_i = \lambda_{T,1}\mathcal{C}_i$ is equivalent to that of Eqs. (1-2) used throughout the paper. From here we could infer that its behaviour applied to a system with more than two networks is also expected to be similar to that of C_A and C_B, and in fact extensive numerical results confirm this statement. In particular, Supplementary Fig. 7 shows that the results obtained with this different definition of the centrality are identical to those obtained for $\mathcal{C}_i$ (compare the plot with Fig. 2 of the main manuscript).

◦ Competition for the betweenness centrality

The betweenness centrality of a node p takes into account the number of shortest paths between any pair of nodes in the network that pass through node p [2, 31]. Nodes with high betweenness centrality have a large influence on the transfer of information through the network, under the assumption that the transfer follows the shortest paths. We can define the betweenness centrality of a network i that competes with other $m-1$ networks as the normalized sum of the betweenness centrality of all its nodes:

$$C''_i = \frac{\sum_{j_i=1}^{N_i} B_{j_i}}{\sum_{k=1}^{m}\sum_{j_k=1}^{N_k} B_{j_k}}\,,$$

where $j_i = 1, ..., N_i$ are all the nodes of network i, and N_i the size of such network (the same stands for j_k and N_k). Note that with this normalization $0 \le C''_i \le 1$.

Supplementary Figure 8 shows the results of the competition for network betweenness among 500 sets of three networks with variable sizes and mean degree (the same sets as in Fig. 2 of the main manuscript). Regardless of the value of the strength of the strong network $\lambda_{A,1}$, there is a coexistence of three pure Nash equilibria: two of them represent the same system (because the links are undirected) and can be understood as global cooperation solutions ($X_1 = \{A \to B,\, B \to C,\, C \to A\}$ (dark orange) and $X_2 = \{A \to C,\, C \to B,\, B \to A\}$ (pale orange)), while the third solution is the already known submission to the strong network X_∞. Unlike the competition for eigenvector centrality, when networks compete for betweenness centrality all solutions are more or less equally beneficial for the different networks, as shown by the fact that the relative centrality variability ΔC among different Nash equilibria is close to zero for all networks (see Supplementary Fig. 8d) and is independent of $\lambda_{A,1}$. The reason is that, due to the modular structure of the network-of-networks, the creation of one or two new connections among

networks affects very slightly the calculation of shortest paths. On the one hand, shortest paths between nodes belonging to the same network are not affected. On the other hand, the shortest path to arrive to nodes belonging to other networks will make use of the already existing shortest paths inside and outside the network with the only addition of a new step: the connector link between networks. This way, the benefit (i.e., enhancement of betweenness centrality) is just local and only affects the connector nodes. Regarding the lack of reciprocal cooperation (double links) among the Nash equilibria, notice that adding a double link instead of a single one does not modify the configuration of the shortest paths between any pair of nodes, since it just reduces the distance of the connector link to $1/2$, which is a small amount compared to the intra-network contributions. Furthermore, there is not a clear relation between the betweenness of a network and its maximum eigenvalue (what we call strength of a network), in contrast to the case of centralities based on, or related to, the eigenvector centrality.

In summary, the pursuit of betweenness centrality does not result in a real conflict among networks, in the sense that (i) the number and configurations of Nash equilibria does not depend on the properties of the networks, and (ii) payoffs obtained by the networks when they connect according to any of their equilibrium strategies are almost equivalent.

◦ Competition for the closeness centrality

The closeness centrality of a node p is another classical measure of node importance that measures the inverse distance of that node to all other nodes $q = 1, ..., N$ in the network:

$$C_{CLO,p} = \frac{1}{\sum_{q=1}^{N} d_{pq}} \, ,$$

where d_{pq} is the shortest distance between nodes p and q.

In the case of m competing networks, the closeness centrality of a network i is equal to the normalized sum of the closeness of all its nodes:

$$C_i''' = \frac{\sum_{j_i=1}^{N_i} C_{CLO,j_i}}{\sum_{k=1}^{m} \sum_{j_k=1}^{N_k} C_{CLO,j_k}} \, ,$$

where $j_i = 1, ..., N_i$ are all the nodes of network i, and N_i the size of such network (the same stands for j_k and N_k). Note that $C_i''' \leq 1$.

We carried out a numerical analysis of the competition for closeness centrality between $m = 3$ networks with at most $l = 1$ link per network. Such analysis revealed a very simple picture in which, regardless of the properties of the networks, only one Nash equilibrium exists. This unique solution is $X_{\infty,CLO} = \{A(0), B \to A, C \to A\}$, and resembles X_∞ in the fact that two *weak* networks connect through single links to the *strong* one. Nevertheless, the analysis of how closeness centrality is distributed in a network-of networks has some drawbacks compared to the eigenvector centrality. First, the *strong* and *weak* networks must be defined based on the closeness centralities of the isolated networks, and do not necessarily coincide with the intuitive idea of strength based on eigenvalues that we used in the rest of this work. Second, a network may have a high closeness just as a consequence of having a lower number of nodes, which makes this kind of measure confusing when comparing the overall closeness of different networks.

From a theoretical point of view, although closeness centrality is a standard way of measuring node centrality, due to the modular structure of the network-of-networks the difference between double and single connector links is residual, as it happened when working with betweenness centrality. Furthermore, the network with larger closeness does not coincide in general with that of larger maximum eigenvalue. Finally, from a practical point of view, competing for closeness does not yield any interesting phenomenology, as only one Nash equilibrium appears.

Competition of networks with random (Erdős-Rényi) topology

Many real-world networks are characterized by a scale-free topology. In accordance, those are the networks we have used to derive the numerical results reported in the main text. Instead, Supplementary Fig. 9 shows the results of the competition among 100 sets of three networks with random (Erdős-Rényi) topology and variable sizes and mean degree. As already seen for scale-free networks, solutions X_0 and X_∞ usually coexist in the range $\lambda_{B,1} < \lambda_{A,1} \leq \lambda_{B\leftrightarrows C,1}$, being the cooperative solution X_0 optimal for weak networks (B and C) and X_∞ optimal for the strong network A. Also, weak networks can induce a transition from X_∞ to X_0, while the opposite is not possible for the strong network A. In sum, network topology (random or scale-free) does not qualitatively affect the phenomenology here described.

Competition with weighted connector links: the appearance of mixed Nash equilibria

Up to now, we have focused on *pure* strategies, that is, those strategies where each competitor can only connect to the rest of competitors through an integer number of links, since they simplify the analysis and interpretation of the results. However, pure strategies are a subset of an infinite number of *mixed* strategies. In a mixed strategy any distribution of the l connector links is allowed for each competitor, being the weight of the links a real number such that the addition of all weights does not exceed the maximum number of links l. Interestingly, mixed strategies add a probabilistic nature to the game that increases the generality of the results.

In this subsection we analyse the game when all competitors can make use of mixed strategies. We show that the introduction of mixed equilibria does not increase the complexity of the Nash equilibria, and in fact yields to the same phenomenology obtained for pure strategies.

◦ Equivalence between the introduction of mixed strategies and a competition with weighted connector links

Here, we prove that the centralities obtained by the competing networks through a set of weighted connector links (with the sum of weights per player equal to an integer constant l) can be approximated at first order by an equally weighted linear combination of $m-1$ different competitions in which the networks compete against each other isolated from the rest through l connector links. The latter is equal to the payoff of a game with mixed strategies, and therefore the results shown here allow us to use mixed Nash equilibria as first order approximations of the solutions of a competition with weighted connector links.

In order to prove this equivalence, let us suppose we have one strong network (network 1) and $m-1$ weak networks (networks $i = 2, 3, ..., m$). The strong network is connected to every weak network i through a single link of weight w_i, such that $\sum_{i=2}^{m} w_i = l$, where $l \geq 1$ is an integer number. The eigenvector $\vec{u}_{T,1}$ determines the outcome of the competition among all m networks, and it can be approximated at first order by

$$\vec{u}'_{T,1} \propto \vec{u}_{A,1} + \sum_{i=2}^{m}[w_i(\sum_{k=1}^{N_i} \frac{\vec{u}_{A,1}\mathbf{P}\vec{u}_{i,k}}{\lambda_{A,1} - \lambda_{i,k}}\vec{u}_{i,k})]\,, \tag{13}$$

where $\lambda_{i,k}$ and $\vec{u}_{i,k}$ are respectively the k-th eigenvalue of network i and its associated eigenvector, N_i is the number of nodes in network i and $\mathbf{P}$ includes the information related to the connector links (see Supplementary Note 2 for more information).

Now, let us verify whether Eq. (13) can be obtained as a linear combination of $m-1$ different competitions in which the strong network 1 competes against each weak network isolated from the rest through l connector links (or a link of weight l). Such linear combination can be expressed as

$$\vec{u}''_{T,1} \propto \sum_{i=2}^{m} \big(\eta_i[\vec{u}_{A,1} + l\big(\sum_{k=1}^{N_i} \frac{\vec{u}_{A,1}\mathbf{P}\vec{u}_{i,k}}{\lambda_{A,1} - \lambda_{i,k}} \vec{u}_{i,k})]\big) \,, \tag{14}$$

where η_i is the i-th coefficient of the linear combination.

As it was already shown in the first subsection of Supplementary Note 3, the eigenvector of a connected system can be approximated as

$$\vec{u}_{T,1} \sim \sum_{i=1}^{m} \mathcal{C}_i \vec{u}_{i,1} \tag{15}$$

where $\mathcal{C}_i$ is the centrality obtained by network i, $\sum_{i=1}^{m} \mathcal{C}_i^2 = 1$, $|\vec{u}_{i,1}| = 1$ for all i, and $|\vec{u}_{T,1}| = 1$. Regarding Eqs. (13) and (14) the conditions that satisfy $\vec{u}'_{T,1} = \vec{u}''_{T,1}$ are

$$\sum_{i=2}^{m} \eta_i = 1$$

and

$$\eta_i = \frac{w_i}{l} \,.$$

Therefore, η_i can be understood as the probability of network 1 of connecting l links to network i and the competition here can be faced as a stochastic process: connecting the competitors through weighted links in a deterministic way is totally equivalent to repeating the competition a large number of times and deciding the strategy following connection probabilities proportional to the weights.

◦ Analysis of the phenomenology when mixed strategies are allowed

A comprehensive numerical study of the mixed Nash equilibria in the case of three networks reveals that mixed solutions (those involving weighted connector links with real –not only integer– weights) are abundant in the range of parameters for which pure solutions X_0 and X_∞ coexist (Supplementary Fig. 10a and b). In order to evaluate if the possibility of choosing mixed strategies affects the players' preferred outcomes, we define the *mixed-vs-pure relative centrality* C_i^* as

$$C_i^* = \frac{C_i - \min\{C_i(X_0), C_i(X_\infty)\}}{\max\{C_i(X_0), C_i(X_\infty)\} - \min\{C_i(X_0), C_i(X_\infty)\}}.$$

For a given mixed equilibrium, the value of C_i^* relates the realized centrality of a player to those that would be obtained under the pure solutions X_0 and X_∞. Specifically, $C^* > 1$ if a network achieves greater centrality with the mixed solution than with its preferred pure solution (whichever it is X_0 or X_∞). As shown in Supplementary Fig. 10c, the centralities obtained by all three networks under mixed solutions are upper– and lower– bounded by the centralities associated to the pure solutions, so that mixed solutions are not optimal for any competitor: weak networks still get their highest centrality with X_0 and the strong network does best with X_∞. Moreover, the possibility of making weighted connector links does not affect the ability of weak networks to induce a transition from X_∞ to X_0.

Competition when more than one connector link is accepted ($l > 1$)

∘ The competitors never distribute their connector links among different targets: a heuristic proof

As we have presented in the former subsection, the space of pure strategies in a game with l available links is a finite subspace of the space of mixed strategies in a single-link game where the total weight of the link equals l. Even if such a finite subspace may still contain some mixed Nash equilibria that correspond to l links distributed among different targets, in a way that each network receives an integer number of links, the natural preference of all competitors for Nash equilibria based on pure strategies presented in the former subsection (see Supplementary Fig. 10c) implies that the competitors never choose those mixed options. The optimal choice for players always corresponds to (i) the extreme (pure) case where all links are targeted to the same network, regardless of how large l is, or (ii) a refusal to connect.

In consequence, the generalization of the phenomenology to larger number of connector links $l > 1$ is straightforward, and surprisingly does not increase the complexity of the analysis: the results are equivalent to those obtained for $l = 1$ (see main text) as far as each connector link is substituted by l links (or a link of weight l). Therefore, the solutions X_0 and X_∞ become $X'_0 = \{A \Rightarrow B, B \Rightarrow\Leftarrow C\}$ and $X'_\infty = \{A(0), B \Rightarrow A, C \Rightarrow A\}$ respectively, where $Y \Rightarrow Z$ represents network Y connected to network Z through l links and $Y \Leftarrow Z$ vice-versa. Similarly, the transition point where X'_0 stops being a solution becomes $\lambda_{A,1} > \lambda_{B\Rightarrow\Leftarrow C,1}$.

Note that the competition when more than one connector link is accepted enhances the chances of cooperation between weak networks: if they are not sufficiently strong to satisfy $\lambda_{B\leftrightarrows C,1} > \lambda_{A,1}$ and therefore the cooperative configuration X_0 is not an equilibrium solution for the $l = 1$ competition, they can still beat the strong network if they connect through the minimum l such that $\lambda_{B\Rightarrow\Leftarrow C,1} > \lambda_{A,1}$. For that situation all the phenomenology studied in this paper would be applicable.

∘ A numerical example: competition when $l = 2$ connector links are accepted

As a clarifying example, Supplementary Fig. 11 shows the solution profiles when two connector links are available to each player (that is, $l = 2$). Each of those links can be targeted to any of the two other networks, and players also have the possibility of making just one link or none. As described in the main text, the number of possible configurations is now much larger than in the simplest case, specifically there are $\binom{m+l-1}{l}^m = 6 \times 6 \times 6 = 216$ different configurations. Under these conditions, there is a critical transition such that for $\lambda_{A,1} > \lambda_{B\rightrightarrows\leftleftarrows C,1}$ only Nash equilibrium $X'_\infty = \{A(0), B \rightrightarrows A, C \rightrightarrows A\}$ exists, while for $\lambda_{A,1} < \lambda_{B\rightrightarrows\leftleftarrows C,1}$ there exists coexistence of X'_∞ and $X'_0 = \{A \rightrightarrows B, B \rightrightarrows\leftleftarrows C\}$. Once again, the weak networks can induce the transition $X'_\infty \to X'_0$ while the strong one cannot do the opposite.

In summary, the results are totally equivalent to what was already studied for $l = 1$ (see Fig. 2 of main text), but substituting each single link by two links (or a link of double weight). Note that this is exactly the case of the second example shown in Supplementary Note 1, where three professional networks describing the collaboration between physicians in Illinois, USA, were studied. The two weak cities were not able to overcome the importance of the largest one making use of only one connector link, but this was possible by connecting through two links ($l = 2$).

Importantly, these results show that even very weak networks (i.e., networks with largest eigenvalues much lower to the strong one) may overcome the strong competitor just by increasing the number of connections between them up to a situation where the X_0 Nash equilibrium arises.

Competition among networks of different abilities: when some competitors can connect through more powerful or a larger number of links than the rest

In some real systems it could happen that (i) the weight w_i of the connector links that a network i can control, or (ii) the available number of such links l_i, are larger than those of the rest of the networks. We

devote this subsection to analyse these two generalizations of the phenomenology.

The reasoning presented in the former subsections of this Supplementary Note proved that, regardless of how large l is, the competitors either connect all their links to a single network or refuse to connect. Therefore, increasing the number of connector links available by a network is identical to increasing the weight of such connector links, and Supplementary Fig. 12 shows a numerical verification of this equality.

Supplementary Figure 13 shows the solution profiles for 3 competitors when one of them has connector links of larger weight than the rest. As both plots in Supplementary Fig. 12 are identical, the results obtained in Supplementary Fig. 13 are totally applicable to the case in which each network i can use a different number l_i of connector links: it clearly yields that the phenomenology is maintained qualitatively unchanged regardless of the weight of the links. Quantitatively, the greater the weight w_A with respect to w_B and w_C, the larger the number of realizations that allow the A-optimal Nash equilibrium $X_\infty = \{A \to B, B \leftrightarrows C\}$. In a similar fashion, the greater the weights w_B or w_C, the larger the number of realizations that allow the B and C-optimal Nash equilibrium $X_0 = \{A \to B, B \leftrightarrows C\}$. Note that the latter is not evident in Figs. 13d-g corresponding to increasing the weight of the weak networks' connector links, and this is due to the fact that the X-axis shows a *normalized* $\lambda_{A,1}$: the real value of the critical point $\lambda_{A,1} = \lambda_{B\leftrightarrows C,1}$ in Figs. 13d-g increases when the weights of the links that connect networks B and C grow (but its position in the plots remains unaltered). In consequence, to obtain a similar fraction of solutions X_∞ when $w_B = 1.5$ or $w_C = 1.5$, the strong network A needs a larger strength $\lambda_{A,1}$ than in the case in which $w_B = 1.2$ or $w_C = 1.2$.

Regardless of the weight of the links, in those cases where X_0 and X_∞ coexist, weak networks can always induce a transition from X_∞ to X_0, while the opposite is again impossible for the strong network.

In summary, increasing the number of links l_i a certain network can make use of, or increasing the weight w_i of such connector links, are equivalent actions, and both increase the fraction of realizations in which its most favourable Nash equilibrium appears. In any case, the phenomenology presented in this manuscript remains unaltered.

Competition among small networks

Another relevant question is whether exists a minimum size for the networks to verify the phenomenology presented here. Supplementary Figure 14 shows the equilibria profiles for the case of 3 random (Erdős-Rényi) competing networks A, B, C when all networks are made of only 10 nodes. As it happened for larger networks, there is a critical transition such that for $\lambda_{A,1} > \lambda_{B\leftrightarrows C,1}$ only Nash equilibrium $X_\infty = \{A(0), B \to A, C \to A\}$ exists, while for $\lambda_{A,1} < \lambda_{B\leftrightarrows C,1}$, X_∞ and $X_0 = \{A \to B, B \leftrightarrows C\}$ coexist. Furthermore, the weak networks can induce the transition $X_\infty \to X_0$ while the strong one cannot, certifying that this example presents exactly the same phenomenology as the one in the main text (see Fig. 2b of the main text), where the networks were two orders of magnitude larger (around 500-1500 nodes per network).

And what would happen if $N \to 1$, that is, when each network competitor is substituted by a single node? In order to study this limit case where the network nature vanishes, the only natural way to define the "strength" of each node is to include auto-loops to every node with weight equal to the node strength –otherwise, all competitors would share the same maximum eigenvalue $\lambda_{A,1} = \lambda_{B,1} = \lambda_{C,1} = 1$–. The phenomenology studied in the paper is partly recovered (see Supplementary Fig. 15), as the same Nash equilibria appear. Two particular cases are plotted, and identical results were obtained for them: $\lambda_{B,1} = \lambda_{C,1} = 4$ and $\lambda_{B,1} = \lambda_{C,1} = 12$. However, the transition in which X_0 disappears is found for very low values of the strong network (around $\lambda_{A,1} = \lambda_{B\leftrightarrow C}$ instead of $\lambda_{A,1} = \lambda_{B\leftrightarrows C,1}$), which means that the competition between single nodes is very beneficial to the strong network.

In summary, the limit case of only 1 node per network strongly hinders the possibility of weak networks to collaborate and overcome the strong one. It is the "network nature" of the competitors what makes the *power of the weak* arise and the phenomenology here presented totally valid.

Competition among 4 networks

Finally, we are going to describe what happens when more than 3 networks compete for centrality. It is clear that the complexity of the system to analyse grows with the number of competitors m, but as we will show here for the case of $m = 4$ the nature of the phenomenology obtained is similar to what was shown for 3 networks.

Supplementary Figure 16 shows the equilibria profiles for the case of 4 competing networks A, B, C and D such that $\lambda_{A,1} > \lambda_{B,1} > \lambda_{C,1} > \lambda_{D,1}$. Note that the main difference with the previous case is that the presence of an additional weak network D may affect the solution profiles of the competition. In this case there are $\binom{m+l-1}{l}^m = 4 \times 4 \times 4 \times 4 = 256$ different configurations, among which three different equilibria control the outcome of the competition: $X''_0 = \{A \to B, B \leftrightarrows C, D \to B\}$ (also variants with $A \to C$ and/or $D \to C$), $X''_\infty = \{A(0), B \to A, C \to A, D \to A\}$, and $X''_{\infty,P} = \{A \leftrightarrows D, B \to A, C \to A\}$. Interestingly, the addition of one competitor leads to the appearance of a new equilibrium, $X''_{\infty,P}$, in which all weak networks connect to the strong one as in X''_∞, but where the strong network connects to the weakest network. X''_∞ and $X''_{\infty,P}$ never coexist in the same realization (because of the Nash condition), and therefore the latter could be understood as a perturbation of the former. In fact, if we group them together as a single type of equilibrium, results are qualitatively similar to the 3-network case.

Depending on the regime, either B and C or A and D control the migration towards more beneficial equilibria. If $\lambda_{A\leftrightarrows D,1} < \lambda_{B\leftrightarrows C,1}$ (i.e. intermediate values of $\lambda_{A,1}$ and/or low values of $\lambda_{D,1}$), networks B and C can induce the transition from X''_∞ and $X''_{\infty,P}$ to X''_0, as they did in the 3-network competition. If $\lambda_{A\leftrightarrows D,1} > \lambda_{B\leftrightarrows C,1}$, a new regime appears in which networks A and D can induce transition from X''_0 to X''_∞ and $X''_{\infty,P}$. Finally, if $\lambda_{A,1} > \lambda_{B\leftrightarrows C,1}$ (i.e. very large values of $\lambda_{A,1}$), only the equilibrium X''_∞ (or sometimes $X''_{\infty,P}$) exists. Also notice that (i) $\lambda_{A\leftrightarrows D,1}$ tends to $\lambda_{A,1}$ for very weak D, thus recovering the limit case of three networks where B and C take control in all multi-stable regimes, and (ii) it is precisely the addition of a weakest network (D) what allows the largest one (A) to gain control in some regimes of the parameters, in contrast to the 3-network case in which A has no control at all.

In summary, regardless of the number of agents in the competition, the strong network remains at the expense of weaker networks' decisions.

Supplementary Note 6. Competition when the connector nodes decide the strategies and vie for their own benefit

Throughout this work the strategies of each competitor have been decided by each network as a whole, in a way that its target is to benefit the network as much as possible. Here we study the case in which the decisions are taken by the connector nodes (i.e. the most central nodes of each network) pursuing their own benefit. First, we will prove that the payoff of each network as a whole is identical to the individual payoff of each of its nodes, up to a first order approximation. Noteworthy, extensive numerical work shows that second order effects may be relevant to the central nodes' optimal strategies, which affect the final configuration of the game under this scenario. Finally, we will study a more general case where the payoff of the connector nodes is a combination of their own importance and the importance of their respective communities. By introducing a *collective incentive* parameter, we will interpolate between both extreme cases and show that, for scale-free networks, small incentives may be enough to recover the phenomenology that characterizes the *power of the weak*.

Analytical results

In this new version of the game, the competitors are the central nodes of each network. The target of each competitor is to maximize its own payoff, calculated as its total importance (or eigenvector centrality).

Let us study the relation between the payoffs obtained by each competitor in both versions of the game. To simplify the explanation, let us suppose that there are only 2 competitors A and B.

As usual, the eigenvector $\vec{u}_{T,1}$ determines the outcome of the competition between networks A and B. The first eigenvector of the total network T formed by the networks A and B after connection can be approximated to first order by

$$\vec{u}_{T,1} \propto \vec{u}_{A,1} + \epsilon \sum_{k=1}^{N_B} \frac{\vec{u}_{A,1}\mathbf{P}\vec{u}_{B,k}}{\lambda_{A,1} - \lambda_{B,k}} \vec{u}_{B,k} + o(\epsilon^2)\,, \tag{16}$$

from where we obtain

$$\vec{u}_{T,1} \sim \frac{\vec{u}_{A,1} + \epsilon \frac{\sum_{cl} (\vec{u}_{A,1})_i (\vec{u}_{B,1})_j}{\lambda_{A,1} - \lambda_{B,1}} \vec{u}_{B,1}}{\sqrt{1 + \epsilon^2 \left(\frac{\sum_{cl} (\vec{u}_{A,1})_i (\vec{u}_{B,1})_j}{\lambda_{A,1} - \lambda_{B,1}} \right)^2}}\,, \tag{17}$$

where $\vec{u}_{T,1}$ has been normalized such that $|\vec{u}_{T,1}| = 1$, and Eq. (17) is an approximation of Eq. (16) where the least relevant terms of the summation have been neglected (see Supplementary Note 2 for more details).

For simplicity, and as in the rest of the Supporting Information, in this analytical treatment we will use the alternative definition of the centrality $\mathcal{C}_i$ (that verifies that $\sum_i^m \mathcal{C}_i^2 = 1$) instead of C_i (that verifies that $\sum_i^m C_i = 1$, see the first subsection of Supplementary Note 3 for a proof of the equivalence of both definitions).

Following Eq. (17), the eigenvector of the connected system can be approximated as

$$\vec{u}_{T,1} \sim \mathcal{C}_{\mathcal{A}} \vec{u}_{A,1} + \mathcal{C}_{\mathcal{B}} \vec{u}_{B,1}\,, \tag{18}$$

where

$$\mathcal{C}_{\mathcal{A}} \sim \frac{1}{\sqrt{1+\left(\epsilon\frac{\sum_{cl}(\vec{u}_{A,1})_i(\vec{u}_{B,1})_j}{\lambda_{A,1}-\lambda_{B,1}}\right)^2}} \tag{19}$$

$$\mathcal{C}_{\mathcal{B}} \sim \frac{\epsilon\frac{\sum_{cl}(\vec{u}_{A,1})_i(\vec{u}_{B,1})_j}{\lambda_{A,1}-\lambda_{B,1}}}{\sqrt{1+\left(\epsilon\frac{\sum_{cl}(\vec{u}_{A,1})_i(\vec{u}_{B,1})_j}{\lambda_{A,1}-\lambda_{B,1}}\right)^2}}\,. \tag{20}$$

In order to obtain the centralities in the new version of the game, we are interested in measuring the relation between the centrality of a single node before and after the connections. From Eqs. (17-20), we obtain that, for any node k of any of both networks,

$$(\vec{u}_{T,1})_{k\in A} \sim \frac{(\vec{u}_{A,1})_k}{\sqrt{1+\epsilon^2\left(\frac{\sum_{cl}(\vec{u}_{A,1})_i(\vec{u}_{B,1})_j}{\lambda_{A,1}-\lambda_{B,1}}\right)^2}} = (\vec{u}_{A,1})_k \cdot \mathcal{C}_{\mathcal{A}}\,,$$

$$(\vec{u}_{T,1})_{k\in B} \sim \frac{\epsilon\frac{\sum_{cl}(\vec{u}_{A,1})_i(\vec{u}_{B,1})_j}{\lambda_{A,1}-\lambda_{B,1}}(\vec{u}_{B,1})_k}{\sqrt{1+\epsilon^2\left(\frac{\sum_{cl}(\vec{u}_{A,1})_i(\vec{u}_{B,1})_j}{\lambda_{A,1}-\lambda_{B,1}}\right)^2}} = (\vec{u}_{B,1})_k \cdot \mathcal{C}_{\mathcal{B}}\,,$$

and therefore

$$\mathcal{C}_{\mathcal{A}}{}^k = \frac{(\vec{u}_{T,1})_{k\in A}}{(\vec{u}_{A,1})_{k\in A}} = \mathcal{C}_{\mathcal{A}},$$

$$\mathcal{C}_{\mathcal{B}}{}^k = \frac{(\vec{u}_{T,1})_{k\in B}}{(\vec{u}_{B,1})_{k\in B}} = \mathcal{C}_{\mathcal{B}}\,.$$

That is, a first order analytical approximation of the phenomenon yields that all nodes in a network increase or decrease their centrality just as the whole network does, and therefore the benefit or loss of a whole network is the same to each of their nodes. From this perspective, we obtain the interesting result that, even if the central nodes (hubs) –or any other node– of the networks were the ones to decide the strategy of the whole network thinking on their own benefit, the phenomenology would be the same as the case in which the whole network vies for the benefit of the whole ensemble. Finally, note that extensive numerical results yield that this result is only valid as far as Eq. (16) can be approximated by Eq. (17).

Numerical results

In Supplementary Fig. 17 we show the solution profile for different competitions in which the payoffs are measured as the connector node's centrality normalized by the sum of the centralities of all the nodes in the network-of-networks. In this case, strategy X_∞ disappears and is substituted by a new strategy $X'_\infty = \{A \to C, B \to A, C \to A\}$, that is, a variation of the traditional X_∞ where the strong network A decides to collaborate with C. Conceptually, the phenomenology is maintained: collaborative strategy X_0 remains and X'_∞ can still be understood as a submission to the strong network, and in fact the regions in which each solution prevail are the same as in the case studied in the paper. Importantly, when both solutions coexist it is not true in general that network C prefers X_0 over X'_∞, which makes the outcome of the competition qualitatively different.

General case: payoff as a combination of individual and collective importance

In the main text and in Supplementary Notes 1-5 we studied competing networks that behave as collective decision makers. As we already mentioned, this is the case if there is a superior entity (government, funding agency, etc.) that promotes and/or coordinates strategies that benefit the community, or if the members of the community share a common objective.

On the contrary, the first two subsections of Supplementary Note 6 were devoted to addressing the case in which the connection strategy is established by the connector nodes pursuing their own benefit. But what happens if the payoff is a mixture of collective and individual interests, as it happens in many real cases? As a first step towards modelling such complex scenarios, we will follow [32] and introduce a new payoff that includes both individual and collective contributions:

$$\Pi_i = \alpha C_i + (1 - \alpha) C_i^k \tag{21}$$

where C_i is the centrality of network i and and C_i^k is the centrality of the connector node $k \in i$. The parameter α can be interpreted as the incentive by which local authorities promote community-oriented strategies, the side benefits that an agent receives from belonging to a better community, or the cooperativity among members of the group. $\alpha = 0$ is the situation in which the payoff is that of the connector node individually (the case studied in the first two subsections of Supplementary Note 6 and Supplementary Fig. 17), and $\alpha = 1$ is the case in which the payoff is that of the whole network (i.e. the case studied throughout the paper).

In order to assess how much results in this general case resemble those described in the main text, we focused on the *power of the weak* and the features that characterize it. Specifically, for each value of the incentive α, we studied the Nash equilibria for more than 100 sets of networks in the range $\lambda_{B\leftrightarrow C} < \lambda_A < \lambda_{B\leftrightarrows C}$ and recorded the fraction of realizations that fulfil: (i) there is coexistence of two stable solutions and (ii) the weak networks can push the whole system to migrate towards their preferred solution, while the strong networks lack the capacity to do the opposite. The results of such analysis are plotted in Supplementary Fig. 18. Notably, relatively small values of α are enough to observe the *power of the weak* phenomenology in scale-free (Barabási-Albert) networks [27]. Even in the case of no collective incentive ($\alpha = 0$), approximately 50% of the realizations lead to an outcome identical to the one observed in the collective decision scenario. In general, we conclude that cooperation between the weak networks and their control of the game is a frequent outcome that can appear at relatively small (or even zero) levels of collective incentive, although the quantitative details significantly depend on the topology of the networks.

Supplementary References

1. Banerjee, A., Chandrasekhar, A.G., Duflo, E. & Jackson, M.O. The diffusion of microfinance. *Science* **341**, 1236498 (2013).

2. Newman, M.E.J. *Networks: An introduction.* Oxford University Press, New York (2010).

3. Hasanov, T., Ozeki, M. & Oka, N. Microcredit risk assessment using crowdsourcing and social networks. *Proc. of the 15th IEEE/ACIS International Conference on Software Engineering, Artificial Intelligence, Networking and Parallel/Distributed Computing* 53-57, Las Vegas, USA (2014).

4. Battiston, S., Farmer, J.D., Flache, A., Garlaschelli, D., Haldane, A.G., Heesterbeek, H., Hommes, C., Jaeger, C., May, R., & Scheffer, M. Complexity theory and financial regulation. *Science*, **351**, 818 (2016).

5. Anand, K., Gai, P., Kapadia, S., Brennan, S. & Willison, M. A network model of financial system resilience. *Journal of Economic Behavior & Organization*, **85**, 219-235 (2013).

6. Minoiu, C. & Reyes, J.A. A network analysis of global banking: 1978–2010. *Journal of Financial Stability*, **9**, 168-184 (2013).

7. Acemoglu, D., Ozdaglar, A. & Tahbaz-Salehi, A. Systemic risk and stability in financial networks. *Working Paper 18727, National Bureau of Economic Research, Cambridge, Massachusetts.*, (2013).

8. Scialabba, N. E. & Hattam, C. (eds.) *Organic agriculture, environment and food security.* Food and Agriculture Organization of the United Nations. Rome, 2002.

9. Coleman, J.S., Katz, E. & Menzel, H. The diffusion of an innovation among physicians. *Sociometry* **20**, 253-270 (1957).

10. Coleman, J.S., Katz, E. & Menzel, H. *Medical Innovation: A Diffusion Study.* Indianapolis: Bobbs-Merrill (1966).

11. Newman, M.E.J. The structure of scientific collaboration networks. *Proc. Natl. Acad. Sci. USA* **98**, 404-409 (2001).

12. Newman, M.E.J. Coauthorship networks and patterns of scientific collaboration. *Proc. Natl. Acad. Sci. USA* **101**, 5200-5205 (2004).

13. Bonacich, P. Factoring and weighting approaches to status scores and clique identification. *J. Math. Soc.* **2**, 113 (1972).

14. Bonacich, P. & Lloyd, P. Eigenvector-like measures of centrality for asymmetric relations. *Social Networks* **23**, 191-201 (2001).

15. https://webofknowledge.com

16. A. Cardillo, J. Gómez-Gardenes, M. Zanin, M. Romance, D. Papo, F. del Pozo & S. Boccaletti. Emergence of network features from multiplexity. *Scientific Reports* **3**, 1344 (2013).

17. DeLaurentis, D., Han, E.-P. & Kotegawa, T. Network-theoretic approach for analyzing connectivity in air transportation networks. *Journal of Aircraft* **45**, 1669-1679 (2008).

18. Crooks, K.R. & Sanjayan, M. (eds.) *Connectivity Conservation.* Cambridge University Press, Cambridge (2006).

19. Urban, D. & Keitt, T. Landscape connectivity: a graph-theoretic perspective. *Ecology* **82**, 1205-1218 (2001).

20. Langville., A.N. & Meyer, C.D. *Google's PageRank and Beyond: The Science of Search Engine Rankings.* Princeton University Press (2006).

21. Senanayake, U., Piraveenan M. & Zomaya, A. The pagerank-index: Going beyond citation counts in quantifying scientific impact of researchers. *PLoS ONE* **10**: e0134794 (2015).

22. www.eigenfactor.org

23. Lohmann, G., Margulies, D.S., Horstmann, A., Pleger, B., Lepsien, J., Goldhahn, D., et al. Eigenvector centrality mapping for analyzing connectivity patterns in fMRI data of the human brain. *PLoS ONE* **5**: e10232 (2010).

24. Seary, A. & Richards, W. Spectral methods for analysing and visualizing networks: An introduction. In K. C. R. Beiger and P. Pattison (Eds.), *Dynamic Social Network Modelling and Analysis*, pp. 209-228. The National Academic Press (2003).

25. Aguirre, J., Papo, D. & Buldú, J.M. Successful strategies for competing networks. *Nat. Phys.* **9**, 230-234 (2013).

26. Aguirre, J., Buldú, J.M. & Manrubia, S.C. Evolutionary dynamics on networks of selectively neutral genotypes: Effects of topology and sequence stability. *Phys. Rev.* E **80**, 066112 (2009).

27. Barabási, A.L. & Albert, R. Emergence of scaling in random networks. *Science* **286**, 509-512 (1999).

28. Aguirre, J., Buldú, J.M., Stich, M. & Manrubia, S.C. Topological structure of the space of phenotypes: The case of RNA neutral networks. *PLoS ONE* **6**, e26324 (2011).

29. Duby, G. *Le Temps Des Cathédrales. L'Art et la Société (980-1420).* Paris: Gallimard (1976).

30. Seta, C. & le Goff, J. (eds.) *La Città e le Mura.* Roma-Bari: Laterza (1989).

31. Freeman, L. A set of measures of centrality based on betweenness. *Sociometry* **40**, 35-41 (1977).

32. Grauwin, S., Bertin, E., Lemoy, R. & Jensen, P. Competition between collective and individual dynamics. *Proc. Natl. Acad. Sci. USA* **106**, 20622-20626 (2009).
